# Rapid Analysis of *Saccharomyces cerevisiae* Genome Rearrangements by Multiplex Ligation–Dependent Probe Amplification

**DOI:** 10.1371/journal.pgen.1002539

**Published:** 2012-03-01

**Authors:** Jason E. Chan, Richard D. Kolodner

**Affiliations:** 1 Bioinformatics and Systems Biology Graduate Program, University of California San Diego, La Jolla, California, United States of America; 2 Ludwig Institute for Cancer Research, Cancer Center and Departments of Medicine and Cellular and Molecular Medicine, Moores–UCSD Cancer Center, School of Medicine, University of California San Diego, La Jolla, California, United States of America; 3 Institute of Genomic Medicine, School of Medicine, University of California San Diego, La Jolla, California, United States of America; The University of North Carolina at Chapel Hill, United States of America

## Abstract

Aneuploidy and gross chromosomal rearrangements (GCRs) can lead to genetic diseases and the development of cancer. We previously demonstrated that introduction of the repetitive retrotransposon *Ty912* onto a nonessential chromosome arm of *Saccharomyces cerevisiae* led to increased genome instability predominantly due to increased rates of formation of monocentric nonreciprocal translocations. In this study, we adapted Multiplex Ligation–dependent Probe Amplification (MLPA) to analyze a large numbers of these GCRs. Using MLPA, we found that the distribution of translocations induced by the presence of *Ty912* in a wild-type strain was nonrandom and that the majority of these translocations were mediated by only six translocation targets on four different chromosomes, even though there were 254 potential Ty-related translocation targets in the *S. cerevisiae* genome. While the majority of *Ty912*-mediated translocations resulted from *RAD52*-dependent recombination, we observed a number of nonreciprocal translocations mediated by *RAD52*-independent recombination between Ty1 elements. The formation of these *RAD52*-independent translocations did not require the Rad51 or Rad59 homologous pairing proteins or the Rad1–Rad10 endonuclease complex that processes branched DNAs during recombination. Finally, we found that defects in *ASF1-RTT109*–dependent acetylation of histone H3 lysine residue 56 (H3K56) resulted in increased accumulation of both GCRs and whole-chromosome duplications, and resulted in aneuploidy that tended to occur simultaneously with GCRs. Overall, we found that MLPA is a versatile technique for the rapid analysis of GCRs and can facilitate the genetic analysis of the pathways that prevent and promote GCRs and aneuploidy.

## Author Summary

In this study we describe an adaptation of Multiplex Ligation–dependent Probe Amplification (MLPA) for use in the study of gross chromosomal rearrangements (GCRs) that occur in *S. cerevisiae* mutants with increased genome instability. Our previous study found that the presence of a *Ty912* element on a nonessential arm of chromosome V resulted in increased rates of non-reciprocal monocentric translocations arising from recombination between the *Ty912* on chromosome V and ectopic Ty elements on other chromosomes. Using MLPA, we observed that the majority of the translocations targeted six different translocation hotspots even though there were at least 254 potential targets for Ty-mediated translocations in the *S. cerevisiae* genome. Most of the observed translocations were formed by *RAD52*-dependent recombination, although we also identified a *RAD52*-independent recombination pathway that promoted the formation of the same types of translocations at lower rates. Finally, we found that defects in the *ASF1-RTT109*–dependent histone H3 lysine 56 (H3K56) acetylation pathway caused increased rates of both Ty-mediated translocations and whole-chromosome duplications (aneuploidy). This aneuploidy often occurred simultaneously with Ty-mediated translocations. Overall, our results demonstrate that MLPA is a rapid, inexpensive method that allows the analysis of the large number of GCRs needed to understand the pathways that suppress or promote genome instability.

## Introduction

Genome stability is important for normal cellular survival and growth. In contrast, genome instability is associated with abnormal cellular growth. For example, tumor cells often contain multiple genome rearrangements and/or exhibit aneuploidy, and such events are thought to contribute to the development and progression of cancer [1]–[4]. Genome rearrangements are also associated with inborn genetic diseases. For instance, copy number changes mediated by segmental duplications are associated with a diversity of genetic diseases [5] and whole chromosome aneuploidy can cause diseases like Down Syndrome [6]. While the association of genome rearrangements and aneuploidy with human genetic disease is well established, the genetic factors that suppress or enhance genome rearrangements and aneuploidy are less well understood.

We previously developed quantitative genetic assays for measuring the rates at which GCRs occur in *Saccharomyces cerevisiae*. These assays, and modified versions of them, select for progeny that lose a nonessential chromosome arm due to a GCR mediated by either non-repetitive [7]–[10], low-copy repeat [9], [11], or high-copy repeat DNA [12] and allow quantitative genetic analysis of the pathways that suppress or promote the formation of GCRs [13]. Genetic studies using these assays have revealed that numerous genes and pathways contribute to genome stability by suppressing the formation of gross chromosomal rearrangements (GCRs) and/or the loss or gain of whole chromosomes [7], [11], [14]–[22]. To fully understand the mechanisms by which GCRs are formed, it is often necessary to determine their structures and sequence their rearrangement breakpoints. However, such analysis of rearranged genomes remains a challenge, particularly due to the large number that must be analyzed to determine the mechanisms by which the GCRs were formed. A number of different techniques have been used to analyze the structure of GCRs including: 1) Pulse Field Gel Electrophoresis (PFGE) to determine the size of rearranged chromosomes [12], [15], [23]–[27]; 2) different methods for PCR amplification and sequencing of rearrangement breakpoints [7], [11], [12], [20]–[23], [26]–[28]; 3) cloning and/or restriction mapping of rearrangement breakpoints [12], [14], [15]; 4) array Comparative Genomic Hybridization (aCGH) that allows for the identification of regions of copy number change but does not provide information about connectivity of the rearranged regions [12], [14], [15], [23], [26], [27]; and 5) next-generation DNA sequencing, which has the potential to provide considerable detail about the structure of genome rearrangements [29]. However, all of these methods fail to scale when analyzing a large number of rearranged genomes, due to either high costs or the tedious natures of the methods. In this study, we adapted a PCR-based method, Multiplex Ligation-dependent Probe Amplification (MLPA), to supplement the analysis of Ty1-mediated GCRs in a manner that scales well in terms of both cost and time.

MLPA is a multiplex ligation-dependent amplification technique that has been used to identify duplications, deletions, and aneuploidy in human cells [30]–[32]. Briefly, multiple pairs of oligonucleotide probes are designed such that each probe in a pair hybridizes next to the other member of the pair at regions of interest in the genome. The total length of each pair of probes is distinct and is used to identify specific regions in the genome on the basis of the length of the final MLPA product. The probes are hybridized to genomic DNA, and then adjacent probes are ligated and amplified using a common pair of fluorescently labeled oligonucleotide primers. Products are separated and their length and fluorescent intensities measured using capillary electrophoresis. Analysis of the fluorescent intensities allows the determination of copy number differences between control and experimental samples. The main advantage of MLPA is its ability to provide copy number variation data for targeted regions in a rapid, inexpensive, and highly parallel manner. While MLPA does not provide the dense genome-wide coverage of aCGH or next-generation sequencing, it can cover multiple regions of interest simultaneously at a density sufficient for many types of genetic studies. In the present study we demonstrate the utility of MLPA for analyzing GCRs by investigating the target site bias of Ty1-mediated GCRs, *RAD52*-independent formation of Ty1-mediated translocations, and aneuploidy induced by deletion of *RTT109*, a gene encoding a histone acetyltransferase.

## Results

### Adaptation of MLPA to identify non-reciprocal translocations

**Figure 1:**
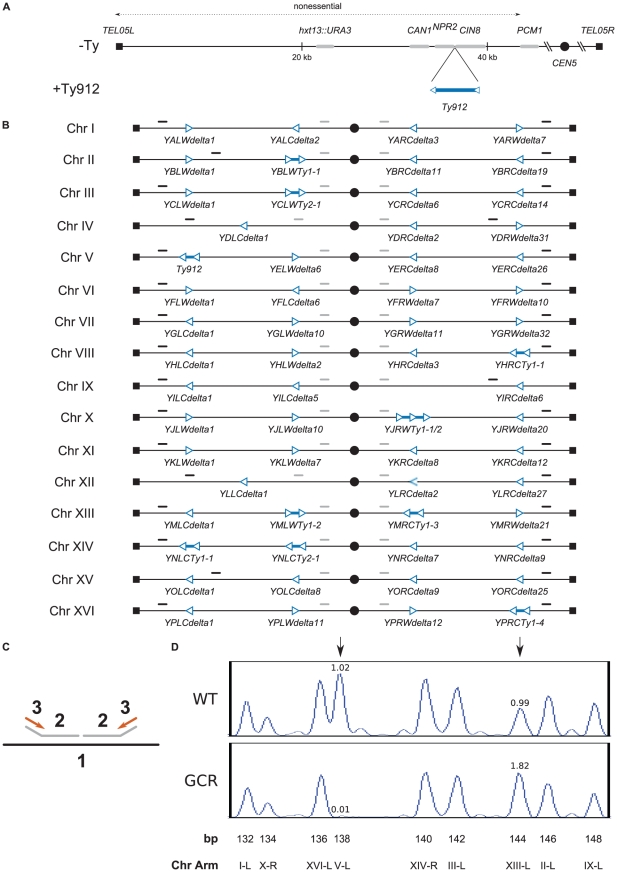
Assay model, design of MLPA telomeric and centromeric probe sets, and verification of results obtained using MLPA. A. Overview of the chromosome V +Ty912 assay strain. The *Ty912* was inserted between *NPR2* and *CIN8* oriented so that it was transcribed towards the telomere. *Ty912* and other genes are not drawn to scale. B. Schematic of the MLPA telomeric and centromeric probes. Telomeric and centromeric probes are designated by solid black and grey lines above the chromosomes, respectively. Ty delta elements are represented as hollow blue triangles, and Ty1 and Ty2 elements are designated as pairs of hollow blue triangles connected by solid blue lines. The orientation of the triangles represent the transcriptional orientation of the elements. Tys and chromosome arms are not drawn to scale. C. Schematic of the MLPA process (1: chromosomal DNA, 2: MLPA probes, 3: universal PCR primers). MLPA probes (2) are hybridized to the chromosomal DNA (1), ligated, amplified by PCR using universal primers (3) and analyzed using capillary electrophoresis. D. Graphical display of MLPA data generated using telomeric MLPA probes. Chromosome arms are represented by peaks in either a wild-type isolate or GCR-containing isolate. Ratios of the normalized peak areas compared to a relative set of wild-type control isolates are given above the indicated peaks of interest. Black vertical arrows indicate either a chromosome V-L deletion (left arrow) or a chromosome XIII-L duplication (right arrow) in the GCR-containing isolate and normal chromosome arm complements in the wild-type isolate.

In previous work we demonstrated that insertion of *Ty912* between *CIN8* and *NPR2* in a nonessential terminal region of chromosome V (the +Ty912 GCR assay) (Figure 1a), led to an increase in the rate of accumulating GCRs [12]. When we screened 88 independent GCR-containing strains derived from either wild-type or one of 11 different mutant strains, we found that all (88 of 88) of these GCR-containing strains contained a deletion of chromosome V from *Ty912* to *TEL05L* (the left telomere of chromosome V) and almost all (82 of 88) of these GCR-containing strains also contained a duplicated region from another chromosome arm bounded by an ectopic Ty1, Ty2, or solo delta sequence at one end and a telomere at the other end. Structural studies demonstrated that these duplication-containing strains each contained a translocation consisting of the centromere-containing fragment of chromosome V joined to the duplicated region of the target chromosome, with a junction involving *Ty912* and the bounding ectopic Ty element of the duplicated region. 94% of the translocations were simple translocations with a single junction involving *Ty912* and a single target ectopic Ty element and 6% appeared to involve a dicentric translocation intermediate that underwent secondary rearrangements. In addition, each GCR-containing strain also contained a wild-type copy of the chromosome from which the duplicated sequence was derived. Overall, this analysis indicated that all of the *Ty912*-mediated translocations observed were formed by a non-reciprocal recombination-mediated translocation mechanism.

To better characterize the distribution of the observed chromosome arm duplications, we developed a MLPA probe set capable of identifying duplicated and deleted chromosome arms. A “telomeric” probe set was designed to identify copy number changes at genomic loci located at the ends of each chromosome arm in *S. cerevisiae*. The probes were designed to hybridize between the telomeres of each chromosome and their closest respective Ty1, Ty2, or solo delta element (Figure 1b, 1c); however, four probes (chrII-L, chrIV-R, chrIX-R, and chrXV-L) could not be designed to meet this criterion due to the lack of suitable non-repetitive DNA regions near the telomeres of these chromosome arms, and were instead designed to hybridize immediately centromeric to the terminal Ty elements present at the 4 chromosome ends (Table S1). As a result, the telomeric probe set theoretically detects 98.4% (250/254) of the possible translocation-associated duplications resulting from *Ty912*-mediated nonreciprocal translocations targeting ectopic Ty-related elements in the S288C reference genome.

Using this telomeric MLPA probe set, we first reanalyzed 5 isolates that were derived from the wild-type +Ty912 GCR assay strain and that were each previously identified by aCGH to contain a translocation chromosome associated with a chromosome arm duplication [12]. The MLPA results concurred with the previous aCGH results and, in each case, identified both the chromosome V-L deletion and the associated chromosome arm duplication identified previously (Figure 1d). We next screened 112 newly isolated independent GCR-containing strains derived from the wild-type +Ty912 GCR assay strain. The MLPA data revealed that all (112 of 112) GCR-containing strains lost the left arm of chromosome V, and almost all (106 of 112) contained a duplicated region of another chromosome arm (Table S2). The remaining isolates either had no detectable duplication (5 of 112) or could not be unambiguously assigned to a rearrangement class (1 of 112).

### Duplication of chromosome arms in the wild-type +Ty912 assay was nonrandom

**Figure 2:**
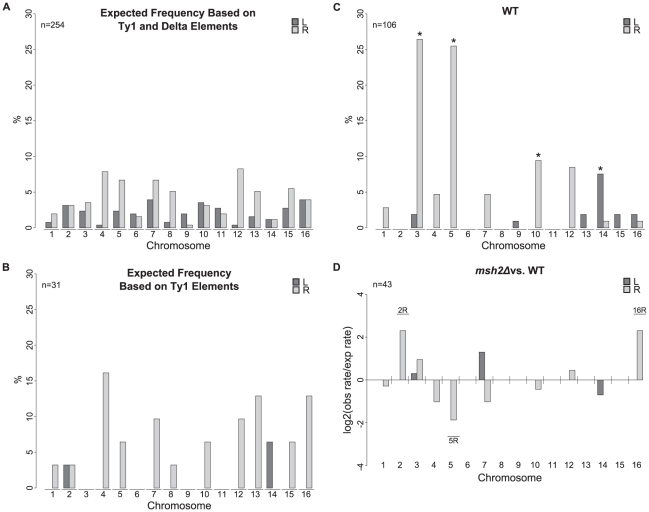
Chromosome arm duplication distributions for wild-type and *msh2Δ* strains versus the distribution of Ty1 and delta elements in the S288C reference genome. A. Distribution of Ty1 and solo delta elements on each chromosome arm. B. Distribution of Ty1 elements on each chromosome arm. C. Observed chromosome arm duplication distribution from 112 wild-type isolates. * indicates significantly overrepresented duplicated chromosome arms compared to the distribution of Ty1 and solo delta elements. D. Log2 ratios of observed versus expected chromosome arm duplication rates from an *msh2Δ* strain compared to wild type. Labeled chromosome arms were duplicated significantly more frequently in the *msh2Δ* mutant than predicted from the bulk increase in GCR rate in the *msh2Δ* mutant compared to the wild-type strain.

We calculated a pair of expected distributions for the chromosome arm duplications based on the assumption that *Ty912* could recombine with either all annotated ectopic Ty1 elements at equal frequency or all annotated ectopic Ty1 and delta elements (in this analysis, each of the 13 Ty2 elements were included as 2 separate delta elements; see Methods) at equal frequency (Figure 2a, 2b). Analysis of the 106 observed duplicated chromosome arms isolated in the wild-type +Ty912 assay strain (Figure 2c; Table S2) revealed numerous chromosome arm duplications that did not contain any annotated full length Ty1 sequences in the S288c reference sequence (chrIII-L, chrIII-R, chrIX-L, chrXIII-L, chrXIV-R, chrXV-L, and chrXVI-L), indicating that, consistent with our previous results [12], both ectopic Ty1 and delta elements are likely to have mediated the observed chromosome arm duplications. We found the distribution of observed chromosome arm duplications to be significantly different from a theoretical distribution that assumed that all Ty1 and delta elements acted as translocation targets (Monte Carlo Sampling of a Multinomial Distribution; 2000 replicates; Empirical p = 5.00×10^−4^). Several chromosome arm duplications were significantly overrepresented in the observed distribution compared to the theoretical distribution, including duplications of chrIII-R (28 times; exact binomial; p = 5×10^−18^), chrV-R (27 times; exact binomial; p = 1×10^−9^), chrXIV-L (8 times; exact binomial; p = 4×10^−5^), and chrX-R (10 times; exact binomial; p = 2×10^−3^). Together these 4 classes of chromosome arm duplications represented 69% (73 of 106) of the observed duplications.

We next sought to identify if there was any bias in this analysis due to the orientation of *Ty912* on chromosome V. We and others have previously shown that translocations targeting centromere-oriented Ty elements yield dicentric chromosomes that must undergo secondary rearrangements to eliminate a centromere [12], [16], [23]. Due to this requirement of secondary rearrangements, these events are likely to be under-represented relative to the proportion of recombinations involving telomere-oriented Ty elements. Thus, it is possible that inclusion of centromere-oriented Ty elements in the list of possible recombination targets biased our analysis.. To check for such bias, we compared the observed distribution of chromosome arm duplications to the distribution of potential telomere-oriented Ty1 and delta element translocation targets (Figure S1). The chrIII-R, chrV-R, chrXIV-L, and chrX-R duplications were still significantly over-represented when compared to this theoretical distribution (exact binomials; p = 2×10^−13^, 1×10^−8^, 4×10^−4^, and 1×10^−2^, respectively). Thus, our MLPA analysis of a large number of GCRs isolated from a single genetic background confirmed the chromosome arm duplication bias we previously noted when we used aCGH to analyze a smaller number of GCR-containing strains isolated from 12 different wild-type and mutant strains [12].

### Identification of individual Ty-related elements mediating recurrent nonreciprocal translocations

**Figure 3:**
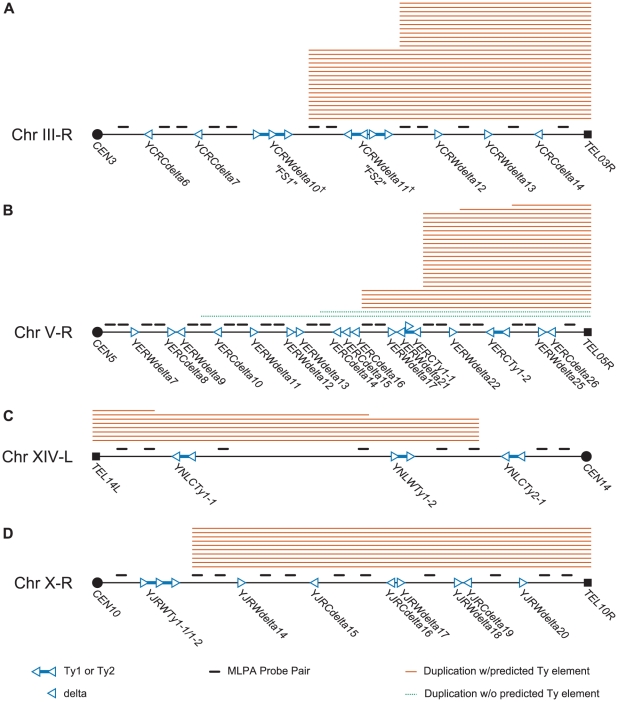
Schematic of MLPA probes designed for hotspot chromosome arms and MLPA data generated from GCR–containing isolates derived from a wild-type strain. Filled squares represent telomeres and filled circles represent centromeres. Hollow triangles represent delta sequences and pairs of hollow triangles connected by blue lines represent Ty1 or Ty2 sequences. The transcriptional orientation of the elements is represented by the direction of the triangles. Solid orange lines above the chromosome arms represent chromosome arm duplications predicted to be mediated by a Ty element; dotted green lines represent duplications predicted to be mediated by a non-Ty element. A. MLPA data for chromosome III-R. Hotspots exist at FS1 and FS2. FS1 represents a tandem pair of Tys replacing the sequence between the 5′ end of *SRD1* to *YCRWdelta10*. FS2 replaces *YCRWdelta11* with an inverted pair of Tys separated by a short spacer sequence. See Ref [15]. B. MLPA data for chromosome V-R. Hotspots exist at or near *YERWdelta17*/*YERWdelta21*/*YERCTy1-1* and the YERCdelta14/*YERCdelta15*/*YERCdelta16* loci. C. MLPA data for chromosome IXV-R. A hotspot exists at the *YNLCTy2-1* Ty2 locus. D. MLPA data for chromosome X-R. A hotspot occurs at the *YJRWTy1-1/YJRWTy1-2* locus.

In order to investigate the possibility that specific Ty-related elements were responsible for the observed chromosome arm duplication bias in the wild-type +Ty912 GCR assay, we created a series of MLPA probe sets specific for chrIII-R, chrV-R, chrX-R, and chrXIV-L. These probe sets contained one or two pairs of primers designed to hybridize between every pair of Ty1, Ty2, solo delta, centromeric, and telomeric element along each chromosome arm, except for a few cases of closely collocated Ty loci (Figure 3a–3d; Tables S3, S5, S6, S7).

We first used the chrIII-R MLPA probe set (Figure 3a; Table S3) to analyze the 28 GCRs involving chromosome III right arm duplications. Two patterns of chromosome arm duplications were observed: those in which the chrIII-R probes telomeric, but not centromeric, to the *YCRWdelta8-YCRWdelta9-YCRWdelta10* locus (61%; n = 17) were duplicated and those in which the chrIII-R probes telomeric, but not centromeric, to the *YCRWdelta11* locus (39%; n = 11) were duplicated (Figure 3a; Table 1). These loci have been previously termed fragile site 1 (FS1) and fragile site 2 (FS2) and were originally identified as hotspots of Ty recombination under conditions of DNA replication stress caused by reduction of the levels of different replicative DNA polymerases [15]. FS1 was found to replace the *SRD1* and *YCRWdelta8-YCRWdelta9-YCRWdelta10* loci in the S288c annotated sequence with a pair of tandem full length Ty1s oriented in a direct repeat orientation. FS2 was found to contain two Ty1s oriented in an inverted head-head configuration with a 283 bp spacer sequence in between the two Ty1s instead of the single annotated *YCRWdelta11* locus. Other studies have also confirmed that these loci show differences from the SGD S288c reference sequence [27], [33]. We confirmed the presence of both the tandem direct repeat pair of full length Ty1s at FS1 and the pair of inverted repeat Ty1s at FS2 in our strains by PCR and sequencing. These results demonstrate that MLPA can be used to identify specific translocation fusion junctions and that FS1 and FS2, even in the presence of normal rates of DNA replication, likely have fragile site activity that results in increased frequencies of Ty-mediated translocations.

**Table 1:** Localization of translocation hotspots by MLPA in a wild-type strain.

Chromosome Arm	Duplicated Probe^a^	Predicted Recombination Target	N^b^	% of Total^c^
III-R	*MAK32 (YCR019W)*	*YCRWdelta8/YCRWdelta9/YCRWdelta10*	17	61%
	*FEN2(YCR028C)*	*YCRWdelta11*	11	39%
V-R	*ICL1 (YER065C)*	*?*	1	4%
	*PMD1 (YER132C)*	?	1	4%
	*GDI1 (YER136W)*	*YERCdelta14/YERCdelta15/YERCdelta16*	5	19%
	*COX15 (YER141W)*	*YERWdelta17/YERWdelta21/YERCTy1-1*	18	67%
	*YER158C*	*YERWdelta22*	1	4%
	*YER163C*	*YERCTy1-2*	1	4%
XIV-L	*CUS2 (YNL286W)*	*YNLCTy1-1*	1	12.5%
	*POR1 (YNL055C)*	*YNLWTy1-2*	1	12.5%
	*NCE103 (YNL036W)*	*YNLCTy2-1*	6	75%
X-R	*YJR030C*	*YJRWTy1-1/YJRWTy1-2*	10	100%

a The most centromeric probe with increased copy number.

b Number of isolates identified with the given recombination target.

c Total is defined as the number of isolates with the indicated chromosome arm duplication.

We next analyzed the GCR-containing strains associated with other overrepresented chromosome arm duplications (chrV-R, XIV-L, and X-R) using the remaining chromosome arm-specific MLPA probe sets (Tables S5, S6, S7). The MLPA data revealed the existence of Ty-mediated translocation hotspots on each of the chromosome arms (Figure 3b–3d; Table 1). The majority of duplications (18 of 27) of the right arm of chromosome V appeared to target the linked *YERWdelta17*, *YERWdelta21*, and *YERCTy1-1* loci (note that this region also contains an unannotated partial Ty sequence [12]), with a smaller proportion (5 of 27) of duplications targeting the region containing the linked *YERCdelta14*, *YERCdelta15*, and *YERCdelta16* loci. This is in agreement with several studies that observed the involvement of the linked *YERWdelta17*, *YERWdelta21*, and *YERCTy1-1* loci in Ty-mediated repair of DNA double strand breaks (DSBs) [9], [26], including our own prior observation of a translocation in which *Ty912* of our assay targeted the centromere-oriented *YERCTy1-1* on chrV-R and resulted in a dicentric translocation chromosome. This dicentric chromosome then underwent additional rounds of rearrangements to yield a monocentric translocation that had duplicated the telomeric end of chrV-R [12]. Likewise, 75% (6 of 8) of the chromosome arm duplications involving the left arm of chromosome XIV appeared to target *YNLCTy2-1*. All of the duplications on the right arm of chromosome X (10 of 10) appeared to involve the *YJRWTy1-1/YJRWTy1-2* tandem Ty1 locus. Overall, these results indicate that 63.2% (67 of 106) of the total Ty-mediated chromosome arm duplications identified in the wild-type +Ty912 assay strain could be accounted for by only 6 Ty target regions on 4 chromosome arms, which contrasts with the presence of 254 potential targets in the *S. cerevisiae* genome.

### An *msh2Δ* mutation does not greatly affect the distribution of chromosome arm duplications

One possible explanation for the observed Ty target bias is that *Ty912* preferentially recombines with regions of high sequence homology. Although this explanation was at odds with the results for chromosome III (*Ty912* has 93% average sequence identity with Ty-related elements on chromosome III-L involved in 2 chromosome arm duplications and 77% average sequence identity with Ty-related elements on chromosome III-R involved in 28 chromosome arm duplications), overall sequence identity may not be the appropriate measure of homology that mediates recombination with *Ty912*, as a previous study examining DSB-induced HR between Ty elements has noted [27]. We therefore investigated the possibility that translocations mediated by recombination between divergent homologous regions are suppressed by mismatch repair [11], [34], [35]. Thus, if sequence homologies drive the target distribution bias seen for Ty-mediated translocations, an *msh2Δ* mutation that eliminates suppression of homeologous recombination should alter the distribution of target Ty elements and chromosome arm duplications.

An *msh2Δ* mutation resulted in an approximately 3-fold increase in the rate of *Ty912*-mediated GCRs (Table 2). All 44 GCRs isolated in the *msh2Δ* mutant were associated with a chromosome V-L deletion and 43 of these GCRs were also associated with a chromosome arm duplication. In order to look for significant changes between the *msh2Δ* strain relative to the wild-type strain, we calculated the fold change between the observed duplication rate of each chromosome arm in the *msh2Δ* mutant relative to an expected duplication rate for each chromosome arm (see Methods). The expected duplication rate assumed that the *msh2Δ* mutation evenly increased the duplication rates of all chromosome arms by the bulk-fold change between the *msh2Δ* mutant and wild-type GCR rates and hence the *msh2Δ* mutation was expected to increase the rate of each duplication 3-fold (Table 2); in other words, we assumed the *msh2Δ* mutation did not preferentially affect the rate of any specific translocation (see Methods). The duplication rates for each chromosome arm in the *msh2Δ* mutant were generally within the 95% confidence interval of the predicted *msh2Δ* rates and were approximately three-fold higher than the wild-type rates, consistent with the idea that the *msh2Δ* mutation increased both the average GCR rate and the translocation rate of each chromosome arm by 3-fold. The only exceptions were the duplication rates of chromosome arms II-R, XVI-R, and V-R. Chromosome arm II-R and XVI-R duplications were not previously seen among the 112 wild-type GCRs analyzed and occurred at about a five-fold higher rate in the *msh2Δ* mutant than predicted from the maximum wild-type rate calculated for these 2 sites. The duplication rate of chromosome V-R was approximately four-fold lower than predicted from the wild-type rate (Figure 2c). The duplication rates of all three of these chromosome arms were outside the 95% confidence interval of their predicted duplication rates calculated from the measured *msh2Δ* bulk rate (p<0.05). In addition, the number of chromosome V duplications observed in the *msh2Δ* mutant was significantly lower than that seen in the wild-type strain (Fisher's Exact Test; p = 0.0122). However, GCRs associated with duplicated chromosome arms containing the translocation hotspots (chrIII-R, V-R, X-R, and XIV-L) were seen in both the *msh2Δ* +Ty912 assay strain and the wild-type +Ty912 assay strain in roughly equal proportions (70% vs. 69%). These results indicate that while an *MSH2*-dependent function may both modestly affect the rate of specific translocations and be potentially important for the formation of translocations involving the chromosome V-R hotspot, mismatch repair is largely not responsible for the biased distribution of translocations in the wild-type +Ty912 assay strain. This supports the idea that the sequence homology relationships between *Ty912* and the other Ty elements in the *S. cerevisiae* genome are unlikely to be the sole, or major, determinant of translocation target site selection.

**Table 2:** GCR rates.

Strain	RDKY	GCR Rate^a^
wild-type	6076	8.4 [5.9–9.6]×10^−8^ (1)^b^
*msh2Δ*	6607/6608	2.2 [0.8–10]×10^−7^ (3)^b^
*rad51Δ*	6555/6556	5.9 [2.5–9.2]×10^−7^ (7)^b^
*rad59Δ*	6599/6600	6.1 [5.0–8.4]×10^−8^ (0.7)^b^
*rad51Δ rad59Δ*	7083/7084	7.9 [5.9–17]×10^−8^ (0.9)^b^
*rad52Δ*	6503/6504	1.3 [0.7–2.5]×10^−8^ (0.2)^b^
*rad52Δ rad51Δ*	7187/7188	1.9 [0.3–5]×10^−8^ (0.2)^b^
*rad52Δ rad59Δ*	7191/7192	7.0 [2.5–17]×10^−9^ (0.1)^b^
*rad52Δ rad51Δ rad59Δ*	7085/7086	1.7 [0.7–4.2]×10^−8^ (0.2)^b^
*rad1Δ*	7356/7357	7.5 [3.4–17]×10^−8^ (0.9)
*rad52Δ rad1Δ*	7358/7359	1.7 [0.4–3.4]×10^−8^ (0.2)
*rtt109Δ*	7492/7493	1.7 [0.9–2.5]×10^−6^ (20)
*rtt109Δ*	7492	1.7 [1.4–1.9]×10^−6^ (20)
*without chromosome duplications*		1.7 [1.3–1.9]×10^−6^ (20)
*with chromosome duplications*		1.9 [1.2–3.3]×10^−6^ (23)
*rtt109Δ* +8	*	2.2 [1.6–3.1]×10^−6^ (27)
*vps75Δ*	7354/7355	7.8 [2.5–17]×10^−8^ (0.9)
*asf1Δ*	6519/6520	1.6 [1.1–3]×10^−6^ (19)^b^
*rlf2Δ/cac1Δ*	7183/7184	1.8 [0.8–3.4]×10^−7^ (2)^b^
*asf1Δ rlf2Δ/cac1Δ*	7352/7353	1.0 [0.5–2.1]×10^−5^ (120)
*hht1-hhf1Δ hht2-hhf2::H3K56G*	7366/7367	8.8 [5.9–15]×10^−7^ (10)
*hht1-hhf1Δ hht2-hhf2::H3K56R*	7364/7365	5.4 [3.4–11]×10^−7^ (6)

a Numbers in brackets represent the 95% confidence interval of the median; numbers in parenthesis represent fold over wild-type.

b See Ref. [12].

*MAT**a** *rtt109Δ* +8 mutants were generated by sporulating from 2n+1 diploid strains RDKY7709-7711.

### Translocation target selection is not associated with physical proximity to *Ty912*

One possible reason for the fact that six hotspots comprise nearly 70% of the observed chromosome duplications is that the six hotspots lie in close physical proximity to *Ty912*, as such physical proximity may determine the ease with which homologous sequences can be targeted by HR. Using previous data that mapped the 3-dimensional spatial relationship of all chromosomes in wild-type cells [36], we analyzed whether any of the six observed hotspots as well as the 20+ kb regions containing each hotspot were in close proximity to the region of chromosome V where *Ty912* was inserted. Analysis of the Chromosome Conformation Capture-on-Chip (4C) data generated with HindIII indicated that 2 of the 6 hotspots showed limited association with *Ty912* on the left arm of chromosome V, whereas analysis of the data generated with EcoRI indicated that none of the hotspots were located adjacent to chrV-L (Table S4). Thus, we found minimal to no interactions between the region surrounding *Ty912* and the observed hotspot locations. Consequently, it is unlikely that physical proximity plays a large role in the selection of the most commonly used translocation targets.

### *rad51Δ* and *rad59Δ* mutations alter the distribution of chromosome duplication targets

Most of the GCRs identified using the +Ty912 assay appear to result from *RAD52*-dependent Ty-mediated HR resulting in non-reciprocal translocations [12]. In *S. cerevisiae*, *RAD52*-dependent HR is primarily mediated by two pathways, which are dependent upon *RAD51* and *RAD59*, respectively [37]. Surprisingly, a *rad51Δ* single mutant had a significantly increased rate of Ty-mediated GCRs compared to wild type (Wilcoxon rank sum test; p = 4.89×10^−7^), whereas a *rad59Δ* mutation decreased the GCR rate of the *rad51Δ* mutant strain to one equivalent to the GCR rate of the wild-type +Ty912 assay strain (Wilcoxon rank sum test; p = 0.89) (Table 2; Table S2). The *rad59* smutation had no significant effect on the Ty-mediated GCR rate of a wild-type strain (Wilcoxon rank sum test; p = 0.34). Interestingly, our previous results indicate that the GCR rate of the *rad51Δ rad59Δ* +Ty912 assay strain was 53-fold higher that that seen for the *rad51Δ rad59Δ* −Ty912 assay strain, suggesting that *Ty912*-mediated GCRs occur at low rates in the *rad51Δ rad59Δ* mutant (Wilcoxon rank sum test; p = 2.09×10^−6^) [12].

**Figure 4:**
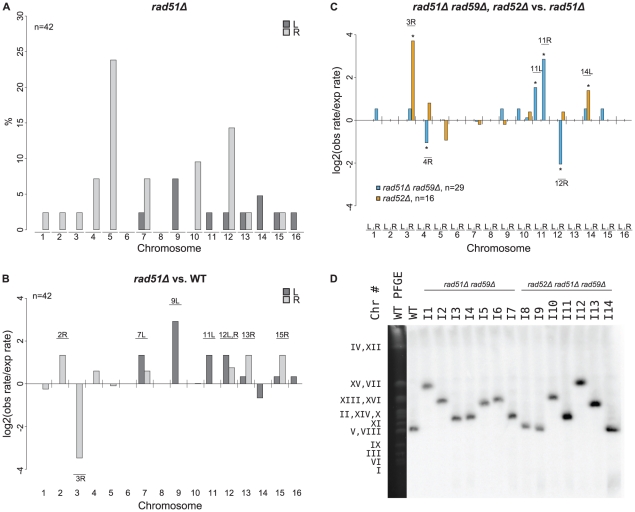
MLPA analysis of GCRs derived in a *rad51Δ* strain. A. Distribution of chromosome arm duplications observed in the *rad51Δ* strain. B. Duplication rates of different chromosome arms from a *rad51Δ* strain compared to that of wild type. Log2 ratios of the observed duplication rates compared to the expected duplication rates are plotted for each chromosome arm. Labeled chromosome arms had significantly increased or decreased rates of duplication in the *rad51Δ* mutant than the bulk increase in GCR rate in the *rad51Δ* mutant compared to the wild type GCR rate. Chromosome III-R appears significantly reduced in the *rad51Δ* mutant strain. C. Comparison of *rad51Δ rad59Δ* and *rad52Δ* mutant strains relative to a *rad51Δ* mutant strain. * represents chromosome arms with significantly increased or decreased rates of duplication for the given strain compared to a *rad51Δ* strain. D. Southern blot of a Pulse Field Gel with separated chromosomes from GCR-containing isolates derived from *rad51Δ rad59Δ* and *rad51Δ rad59Δ rad52Δ* mutant strains reveals chromosome Vs that are larger than wild type (I1–I7 and I10–I13). *MCM3*, an essential gene on chromosome V, was used as the probe.

To investigate how a *rad51Δ* mutation affected the formation of GCRs, we analyzed 46 GCR-containing isolates derived from a *rad51Δ* +Ty912 assay strain (Table S2). All 46 isolates contained a chromosome V-L deletion, and 42 of these isolates each contained a chromosome arm duplication (Figure 4a). Four isolates did not have any accompanying chromosome arm duplications, which was a small, but statistically significant, increase compared to the bulk fold increase in the total GCR rate (p<0.05). This suggests that a *rad51Δ* mutation results in a small increase in the rate of formation of broken chromosomes that are subsequently healed by *de novo* telomere addition [19]. There was a statistically significant decrease in the proportion of GCRs associated with chromosome III-R duplications arising from the *rad51Δ* mutant compared to a wild-type strain (Fisher's exact test; p = 4.16×10^−4^) and, using the same method that was used to analyze the effect of an *msh2Δ* mutation on the rate of individual chromosome arm duplications, we found that the rate of chromosome III-R duplications was significantly decreased as well (p<0.05; Figure 4b). In contrast, the rates of the chromosome arm duplications involving the chromosome V-R, X-R, and XIV-L hotspots were not preferentially affected by the *rad51Δ* mutation. Additionally, chromosome IX-L and XII-R duplications were statistically significantly increased in the *rad51Δ* mutant compared to the relative bulk effect of a *rad51Δ* mutation. There were also statistically significant increases in the rates of duplication of several chromosome arms not originally observed among the 112 wild-type derived *Ty912*-mediated GCRs (chromosomes II-R, VII-L, XI-L, XII-L, XIII-R, and XV-R) (Figure 4b; p<0.05). Overall, these results suggest that *RAD51* is important for the formation of GCRs involving the hotspots on chromosome III-R and is important for the suppression of GCRs involving many other Ty-related translocation targets.

To better understand the function of *RAD59* in promoting GCRs in a *rad51Δ* mutant, we analyzed 30 *Ty912*-mediated GCRs obtained from a *rad51Δ rad59Δ* double mutant (Table S2). We did not analyze a large number of GCRs isolated from a *rad59Δ* single mutant as a *rad59Δ* mutation did not significantly change the GCR rate in the +Ty912 assay (Wilcoxon rank sum test; p = 0.343) (Table 2). All 30 GCRs derived from the *rad51Δ rad59Δ* double mutant contained the chromosome V-L deletion and 29 of these GCRs were also associated with single chromosome arm duplications. Compared to the bulk fold increase in the GCR rate relative to the wild-type GCR rate, the *rad51Δ rad59Δ* double mutant had statistically significant decreases in the duplication rates of chromosomes III-R, IV-R, and XII-R, and an increase in the duplication rate of chromosome IX-L (p<0.05). We also noted chromosome X-L (n = 1), chromosome XI-L (n = 2) and chromosome XI-R (n = 5) duplications, which were not seen in the wild-type chromosome arm distribution. The *rad51Δ rad59Δ* double mutant had a lower overall GCR rate than the *rad51Δ* single mutant (Wilcoxon rank sum test; p = 1.13×10^−5^) (Table 2). Compared to the bulk affect of a *rad59Δ* mutation in a *rad51Δ* single mutant (see Methods), the *rad51Δ rad59Δ* double mutant had decreased rates of duplication of chromosome arms IV-R and XII-R, as well as increased rates of duplication of chromosome arms XI-L and XI-R (Figure 4c; p<0.05). In addition, the duplication rates of the chromosome arms associated with the chromosome III-R, V-R, X-R and XIV-L hotspots were reduced in the *rad51Δ rad59Δ* double mutant relative to the *rad51Δ* single mutant to the same extent as the bulk GCR rate (Figure 4c, p>0.05). Overall, these results support the view that a *RAD51*-dependent HR pathway is important for suppressing *Ty912*-mediated translocations and that most of the translocations seen in the *rad51Δ* single mutant are promoted by *RAD59*-dependent HR.

To verify that the duplicated chromosome arms associated with the GCRs formed in the *rad51Δ rad59Δ* double mutant were indeed fused to chromosome V, we analyzed 7 *rad51Δ rad59Δ*-derived GCRs using PFGE followed by Southern blotting with a probe specific to *MCM3*, an essential gene on chromosome V. The results showed that all 7 GCRs (I1–I7) were associated with abnormally large chromosome Vs, consistent with the duplicated chromosome arms being fused to deleted chromosome Vs (Figure 4d), and reminiscent of previously investigated nonreciprocal translocations [12]. We did not verify the nature of the fusion junctions in these translocation chromosomes, although, as discussed below, we did verify the nature of several such translocation junctions identified in *rad52Δ* single and *rad52Δ rad51Δ rad59Δ* triple mutants.

### *RAD52*-independent GCRs can be formed by HR

**Figure 5:**
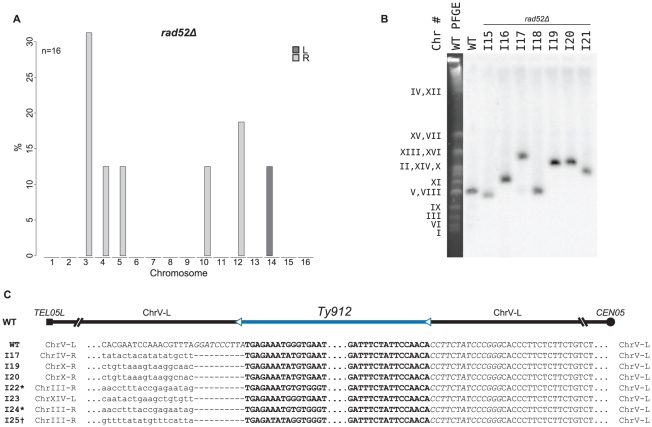
MLPA, Southern blot, and sequencing analyses of GCRs derived from a *rad52Δ* recombination-deficient mutant strain. A. MLPA data reveals duplicated chromosome arms associated with GCRs derived in a *rad52Δ* mutant strain. B. Southern blot of a Pulse Field Gel with separated chromosomes from GCR-containing isolates from a *rad52Δ* mutant strain also reveals larger than wild-type chromosome Vs (I16, I17, and I19–I21). C. Pseudo multiple sequence alignment of sequenced breakpoint junctions from *rad52Δ* derived GCRs. Italicized base pairs flanking *Ty912* on chromosome V represent nonreference-sequence restriction sites used for cloning and inserting *Ty912* on chromosome V. * - sequence upstream of the 5′ end of the Ty- element mediating the translocation matches part of the reference sequence supposedly deleted by FS2; this suggests that the FS2 loci is not completely identical to that described originally in [15]. † Amplified fragment is consistent with two tandem Tys in FS1; however, the 5′ flanking sequence displayed is part of the tandem Ty1 construct and was what was read when the 5′ primer used to amplify the fragment was used to sequence the fragment.

There was a 5-fold decrease in the GCR rate of the *rad52Δ* +Ty912 assay strain compared to that of the wild-type +Ty912 assay strain (Wilcoxon rank sum test; p = 1.69×10^−05^), consistent with the bulk of the *Ty912*-mediated GCRs being formed by HR (Table 2). The GCR rate of the *rad52Δ* +Ty912 assay strain, however, was previously determined to be slightly, but not strictly significantly, higher than the GCR rate of the *rad52Δ* −Ty assay strain (Wilcoxon rank sum test; p = 0.0625) [12], suggesting that *Ty912*-mediated GCRs may still occur at low rates in the *rad52Δ* +Ty912 assay strain. To further investigate the role of *RAD52* in the formation of Ty1-mediated GCRs, we used MLPA to analyze 50 independent GCRs derived from a *rad52Δ* +Ty912 assay strain (Table S2). All 50 GCRs were associated with the chromosome V-L deletion. We did not detect any additional genome alterations in 34 (68%) of these isolates, a finding consistent with a high rate of GCRs mediated by *de novo* telomere additions in a *rad52Δ* mutant [11], [19], [38]. In 16 (32%) of the isolates, we observed additional chromosome arm duplications, with each isolate containing one chromosome arm duplication (Figure 5a). This represented a significant decrease in the proportion of GCRs associated with chromosome arm duplications when compared those obtained from a wild-type strain (Fisher's Exact test; p = <2.2×10^−16^). Interestingly, the *rad52Δ* mutant shared none of the significant chromosome arm duplication changes seen in the *rad51Δ rad59Δ* double mutant when these two mutant strains were compared with a *rad51Δ* single mutant, although this may be due to the small number of chromosome arm duplications analyzed in the case of the *rad52Δ* mutant (Figure 4c).

To confirm that the observed chromosome arm duplications seen in the *rad52Δ* mutant were associated with the formation of nonreciprocal translocations involving chromosome V-L and the duplicated chromosome arms, we further analyzed 7 *rad52Δ*-derived GCR-containing isolates, two of which (I15 and I18) did not have chromosome arm duplications as determined by MLPA and five of which had single arm duplications (I16 [chrIII-R], I17 [chrIV-R], I19 [chrX-R], I20 [chrX-R], and I21 [chrV-R]). Analysis of these isolates by PFGE followed by Southern blotting utilizing a probe to the chromosome V gene *MCM3* revealed that each of the isolates without chromosome arm duplications contained a smaller than wild-type chromosome V (consistent with the known chromosome V-L deletion associated with a *de novo* telomere addition) and that the isolates with chromosome arm duplications each had a larger than wild-type chromosome V (Figure 5b). No other chromosomes appeared to have altered lengths. Analysis of these same 7 GCR-containing strains by aCGH revealed that all 7 had a deletion of the left arm of chromosome V between *TEL05L* and *Ty912* (Figure S2). Additionally, each of the 5 strains identified by MLPA to contain a chromosome arm duplication had an additional duplicated region of DNA bounded by a full length Ty1 or solo delta element at one end and a telomere at the other end (Figure S2; Table S8); the amplified chromosome arms identified by MLPA were the same as those identified by aCGH. PCR amplification of the breakpoints of 3 of the GCRs (I17, I19 and I20) associated with chromosome arm duplications confirmed that these GCRs were the result of a fusion between chromosome V-L at *Ty912* and the Ty or delta element bounding the duplicated region associated with each GCR (Figure 5c; Table S8); the other 2 breakpoints could not be amplified. In addition, PCR amplification and sequencing of the breakpoints of four independent translocations (I22–25) occurring in the *rad52Δ* +Ty912 assay strain and identified by MLPA to be associated with a chromosome arm duplication similarly demonstrated the presence of translocation chromosomes that resulted from fusions between chromosome V-L at *Ty912* and the Ty or delta elements bounding the duplicated region associated with each GCR (Figure 5c; Table S8). Thus, deletion of *RAD52* decreases, but does not completely eliminate, HR that promotes Ty-mediated nonreciprocal translocations [19], [38].

**Figure 6:**
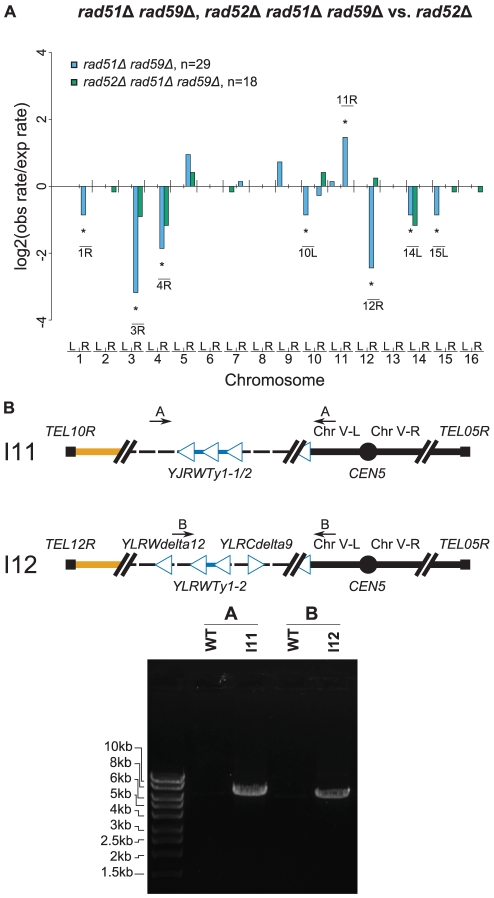
Further MLPA and PCR analyses of GCRs derived from recombination-deficient mutant strains. A. Comparison of chromosome arm duplication rates in various HR deficient strains to those from a *rad52Δ* mutant strain. The *rad51Δ rad59Δ rad52Δ* distribution was almost identical to the *rad52Δ* distribution, indicating that the *rad52Δ* mutation was epistatic to the *rad51Δ rad59Δ* mutations. B. PCR amplification of the breakpoint junction from two *rad51Δ rad59Δ rad52Δ* GCR-containing isolates (I11 & I12). Primer pairs used were A (JCP392 & JCP310) and B (JCP670 & JCP310) [12].

We next examined whether *RAD51* and *RAD59*, which encode homologous pairing proteins [37], and the Rad1–Rad10 complex, which processes non-homologous single-stranded DNA tails that form during single-stranded annealing [39], were required for the formation of *Ty912*-mediated translocations formed by *RAD52*–independent HR. To this end, we investigated to differing degrees the rate and structure of *Ty912*-mediated GCRs in *rad52Δ* and *rad52Δ rad59Δ* double mutants, the *rad52Δ rad51Δ rad59Δ* triple mutant, and the *rad52Δ* double mutant. All four mutant strains had GCR rates that were statistically similar to the GCR rate of a *rad52Δ* single mutant (Table 2) (Wilcoxon rank sum test; p = 0.21, 0.10, 0.95 and 0.79, respectively) and had statistically similar frequencies of GCRs containing and lacking chromosome arm duplications (Fisher's Exact Test with Bonferroni correction for multiple hypothesis testing; all p values>0.0167) (Figure 6a; Figure S3; Table S2) [40]. The chromosome arm duplication patterns of the *rad52Δ* double and triple mutants were also highly similar to that of the *rad52Δ* single mutant (Figure 6a; Figure S3), unlike that of the *rad51Δ rad59Δ* double mutant (see above).

To confirm that the chromosome arm duplications seen in the *rad52Δ rad51Δ rad59Δ* mutant were associated with the formation of nonreciprocal translocations involving chromosome V-L and the duplicated chromosome arms, we analyzed 7 GCRs isolated from this mutant by PFGE followed by a Southern blotting utilizing a probe to *MCM3*. Four GCRs were shown by MLPA to be associated with single chromosome arm duplications (I10 [chrIV-R], I11 [chrX-R], I12 [chr-XIIR], I13 [chrXII-R]) and three were not associated with a chromosome arm duplication (I8, I9, I14) (Figure 4d). The results revealed that the four GCRs associated with chromosome arm duplications (I10, I11, I12, I13) were each associated with an abnormally large chromosome V and the three GCRs that did not involve a chromosome arm duplication (I8, I9, I14) were each associated with a smaller than wild-type chromosome V. The smaller than wild-type chromosome Vs were consistent with the formation of GCRs mediated by *de novo* telomere addition. No other chromosomes appeared to be altered in these 7 GCR-containing strains. PCR amplification of the breakpoints of 2 of the GCRs derived in the *rad52Δ rad51Δ rad59Δ* +Ty912 assay strain (I11 & I12) confirmed the presence of translocation chromosomes that were the result of a fusion between chromosome V-L at *Ty912* and the Ty1 element bounding the duplicated region associated with each GCR (Figure 6b). Thus, deletion of *RAD51* and *RAD59* in a *rad52Δ* mutant does not completely eliminate HR that promotes Ty-mediated nonreciprocal translocations.

#### *rtt109Δ* mutants have increased rates of aneuploidy and *Ty912*-mediated GCRs

Rtt109 is a histone acetyltransferase that acetylates lysine 56 on histone H3 [41], [42] and is thought to be important for DNA repair and DNA damage responses [43], [44]. We previously observed that an *rtt109Δ* mutation increased the rate of *Ty912*-mediated GCRs 20-fold relative to wild type (Table 2) and we also observed whole chromosome duplications among the GCR-containing isolates studied [12]. To further analyze such whole chromosome duplications, we created a “centromeric” MLPA probe set by designing primer pairs that hybridized on either side of the centromere of each *S. cerevisiae* chromosome and that were centromeric to the first Ty1 and solo delta element on each chromosome arm (Figure 1b; Table S9). The telomeric and centromeric MLPA probe sets were then used to determine the incidence of whole chromosome duplications in an *rtt109Δ* mutant, with whole chromosome duplications being defined as the simultaneous duplication of all of the probed regions along a chromosome. We screened 49 independent *rtt109Δ* mutants grown on GCR selective media and found that 11 of the mutants contained whole chromosome duplications (Table S2; Table S10). In addition, we observed that 2 out of 49 *rtt109Δ* mutants grown on nonselective media had whole chromosome duplications (Table S10).

**Figure 7:**
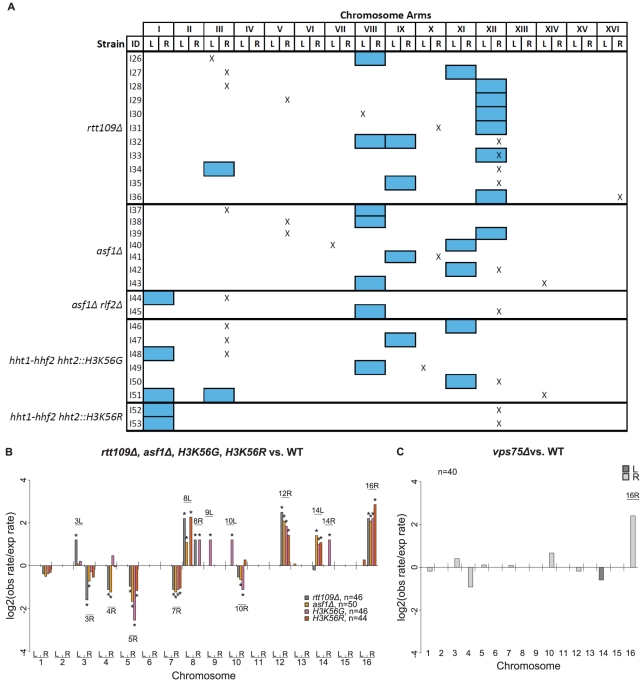
Analysis of GCRs isolated in *rtt109Δ* and related mutants. A. Distribution of chromosome arm duplications. Aneuploidy appears to be uncorrelated with specific GCRs. “X” represents chromosome arm duplications. Blue boxes represent whole chromosome duplications. Note that all strains had a terminal deletion of the left arm of chromosome V (not depicted). B. Log2 ratios between observed and expected chromosome arm duplication rates for mutants deficient for H3K56 acetylation. C. Comparison of *vps75Δ* and a wild-type strain. The *vps75Δ* mutation appears to have little effect on the distribution of GCRs compared to wild type.

In order to measure the correlation between GCRs and whole chromosome duplications in an *rtt109Δ* mutant, we performed a fluctuation test to measure the GCR rate of an *rtt109Δ* mutant using 49 independent cultures. From these 49 independent cultures, we obtained 49 pairs of matched isolates consisting of one single colony grown on the nonselective media and one single GCR-containing colony grown on the GCR-selecting media. We observed that 11 of the 49 GCR-containing isolates had an abnormal whole chromosome count (Figure 7a; Tables S2 and S10). This was a significant difference compared to the 112 independent wild-type strains grown on GCR selective media, none of which had any whole chromosome duplications (Fisher's Exact test; p = 8.75×10^−7^). Of these 11 isolates, 10 contained single whole chromosome duplications, while 1 isolate contained extra copies of two different chromosomes (chromosomes VIII and IX). In contrast to the set of GCR-containing isolates, we identified only 2 isolates among the 49 independent isolates picked from the nonselective media with whole chromosome duplications (Table S10). These 2 isolates did not contain GCRs and the observed whole chromosome duplications were not detected in their matched GCR-containing isolates. These results suggest that an *rtt109Δ* mutation increases the rate of accumulating whole chromosome duplications, consistent with prior observations that an *rtt109Δ* mutant had increased rates of chromosome loss [45]. The results also indicate that there was a statistically significant association of whole chromosome duplications with the presence of GCRs (Exact binomial substitute for McNemar's Test; p = 0.0225). However, the GCR rate calculated using the 11 cultures containing whole chromosome duplications was not statistically different from the GCR rate calculated using the other 38 cultures lacking whole chromosome duplications (Wilcoxon rank sum test; p = 0.871) (Table 2), indicating that the association between GCRs and whole chromosome duplications was likely not due to genetic differences between these two groups of isolates.

It has been suggested that aneuploidy can result in increased genome instability [46]. To determine if aneuploidy causes increased GCR rates, we created aneuploid *rtt109Δ* haploids by first crossing an *rtt109Δ* haploid mutant containing a chromosome VIII duplication with a wild-type haploid strain of the opposite mating type and then sporulating and genotyping the resultant haploids. Of the spore clones containing the *rtt109Δ* mutation and the markers required for the GCR assay, 71.4% (15/21) contained a duplicated chromosome VIII; additionally, 14.3% (3/21) lacked the chromosome VIII duplication and instead had a chromosome XII duplication, and 4.8% (1/21) lacked the chromosome VIII duplication but instead contained a duplicated chromosome IX (Table S11). There was no significant difference in the GCR rates of an *rtt109Δ* mutant containing a duplicated chromosome VIII and an *rtt109Δ* mutant with a normal complement of chromosomes (Wilcoxon rank sum test; p = 0.141) (Table 2), indicating that the chromosome VIII duplication did not alter the GCR rate. This is consistent with the observation above that the group of matched cultures with and without whole chromosome duplications had the same GCR rate. Therefore, it seems unlikely that aneuploidy in the *rtt109Δ* mutant increased the rate of accumulating GCRs. To determine if the presence of a GCR increased the rate of accumulating whole chromosome duplications, we used MLPA to screen independent colonies from 3 different strains for the presence of whole chromosome duplications: 1) an *rtt109la*mutant without detectable GCRs, 2) an *rtt109Δ* mutant containing a chrX-R duplication GCR (one of the recurrent GCRs seen in a wild-type strain), and 3) an *rtt109Δ* mutant containing a chrXII-R duplication GCR (the most frequent GCR seen among the 11 *rtt109Δ* aneuploids (Figure 7a)). The number of whole chromosome duplications observed for these 3 mutant strains were 2 out of 49 colonies, 1 out of 48 colonies, and 1 out of 50 colonies analyzed, respectively. The frequencies of aneuploidy in the *rtt109Δ* mutant strains with either a chrX-R duplication or a chrXII-R duplication were not statistically different from that observed in an *rtt109Δ* mutant strain lacking a GCR (Fisher's Exact test; p = 1 and 0.617, respectively). These results indicate that the presence of a GCR in an *rtt109Δ* mutant did not increase the rate of accumulating whole chromosome duplications.

We also used MLPA to analyze the distribution of the chromosome arm duplications present in the 49 GCR-containing strains derived from the *rtt109Δ* mutant (Tables S2 and S10) (see Methods). All 49 isolates contained chromosome V-L deletions associated with single chromosome arm duplications. Compared to the bulk increase in the GCR rate caused by the *rtt109Δ* mutation relative to that of the wild-type strain, the *rtt109Δ* mutant increased the relative rates of chromosome III-L, VIII-L, XII-R, and XVI-R duplications (Figure 7b; Table S2). Of the chromosome arm duplications involving known translocation hotspots, chromosome III-R and V-R duplications had decreased relative rates in the *rtt109Δ* mutant whereas the increases in the duplication rates of chromosomes X-R and XIV-L were the same as the bulk increase in GCR rate of the *rtt109Δ* mutant relative to wild type. We also observed chromosome VIII-L and VIII-R arm duplications that were not seen in the wild-type strain. In contrast, chromosomes III-R, IV-R, V-R, and VII-R duplications were all seen in the wild-type strain and occurred at a reduced rate in the *rtt109Δ* mutant. Overall, these results suggest that *RTT109* is important for the suppression of *Ty912*-mediated GCRs and that suppression of translocations mediated by certain target sites, notably those involved in chromosome III-L, VIII-L, VIII-R, XII-R, and XVI-R duplications, are more dependent on Rtt109 than other targets.

### Aneuploidy occurs due to loss of *ASF1-RTT109*–dependent H3K56 acetylation

We next investigated the potential role of two different pathways involving Rtt109 in suppressing GCRs and aneuploidy. Both Vps75, a histone chaperone, and Asf1, a nucleosome assembly factor, form separate complexes with Rtt109 that acetylate H3K56 *in vitro*, although only the Asf1-Rtt109 complex appears to be required for acetylation of H3K56 *in vivo* [47]. We found that a *vps75Δ* single mutant had essentially the same rate of *Ty912*-mediated GCRs as the wild-type strain, whereas an *asf1Δ* single mutant had an increased rate of *Ty912*-mediated GCRs similar to that seen for a *rtt109Δ* mutant (Table 2). Analysis of 45 *vps75Δ* and 55 *asf1Δ* derived GCR-containing isolates for whole chromosome duplications revealed no whole chromosome duplications among the *vps75Δ*-derived GCR-containing isolates and 7 instances of *asf1Δ*-derived GCR-containing isolates containing whole chromosome duplications (Figure 7a; Table S2). The frequency of whole chromosome duplications in the *asf1Δ* mutant was not significantly different from that of an *rtt109Δ* mutant (Fisher's Exact test; p = 0.207). Additionally, the distribution of chromosome arm duplications seen for the GCRs derived from the *vps75Δ* mutant (40/45 GCRs) was strikingly similar to that seen with the wild-type strain, while the distribution of chromosome arm duplications seen for the GCRs derived from the *asf1Δ* mutant (50/55 GCRs) more closely reflected that of the *rtt109Δ* mutant (Figure 7b, 7c; Table S2).

*RLF2* (also known as *CAC1*) encodes a subunit of CAF-1, a chromatin assembly complex that mediates nucleosome assembly in cooperation with *ASF1* [48]. Consistent with this, an *asf1Δ* double mutant was previously demonstrated to have a synergistic increase in the rate of single copy sequence-mediated GCRs compared to both *rlf2Δ* and *asf1Δ* single mutants [49]. The *rlf2Δ* single mutant strain was similar to the wild-type strain and had only a small 2-fold increase in the *Ty912*-mediated GCR rate (Table 2). When we analyzed 46 *rlf2Δ* derived GCR-containing isolates by MLPA, we detected no whole chromosome duplications and a chromosome arm duplication pattern similar to that seen in the wild-type strain, except for increased rates of chromosome III-L, IX-L, XI-L, and XVI-R arm duplications (Figure S4; Table S2). Similarly, there was no significant increase in the frequency of whole chromosome duplications in an *asf1Δ rlf2Δ* double mutant compared to an *asf1Δ* single mutant (Fisher's Exact test; p = 0.483) (Figure 7a), even though the double mutant had a synergistic 120-fold increase in the GCR rate compared to the *asf1Δ* and *rlf2Δ* single mutants (Table 2). Moreover, 75% (9/12) of the chromosome arm duplications seen in the *asf1Δ rlf2Δ* mutant (29/30 GCRs) were seen in an *asf1Δ* mutant, while the remaining 25% (3/12) chromosome arm duplications were observed in the profile of an *rlf2Δ* mutant. Thus, despite the synergistic increase in GCR rate observed in an *asf1Δ rlf2Δ* mutant, the distribution of chromosome arm duplications appeared to be additive, with the increase in aneuploidy likely a result of only the *asf1Δ* mutation. Taken together, these results suggest that the *rtt109Δ*-dependent increase in whole chromosome duplications is linked to defects in the *ASF1-RTT109*-dependent H3K56 acetylation pathway.

### Acetylation of histone H3K56 suppresses aneuploidy

To investigate whether aneuploidy results from the inability of the *ASF1-RTT109* complex to acetylate histone H3K56, we tested mutations that altered histone H3K56 for their effect on the accumulation of whole chromosome duplications. For this analysis, we constructed strains with chromosomal *hht1-hhf1Δ hht2-hhf2Δ* double deletions carrying plasmids encoding various unacetylatable mutant alleles of *HHT2* and a wild-type copy of *HHF2*. In this manner, we were able to construct *hht1-hhf1Δ hht2-hhf2Δ HHF2 hht2::H3K56G* and *hht1-hhf1Δ hht2-hhf2Δ HHF2 hht2::H3K56R* mutant strains. The strain containing the *hht2::H3K56G* mutation and the strain containing the *hht2::H3K56R* mutation both exhibited increased GCR rates that were slightly lower than that of an *rtt109Δ* mutant (Table 2). All (46/46) and 97.8% (44/45) of the GCR-containing isolates derived from the *hht2::H3K56G* mutant and *hht2::H3K56R* mutant, respectively, were associated with single chromosome arm duplications; there were 6 and 2 isolates of the *hht2::H3K56G* and *hht2::H3K56R* mutant strains, respectively, that had both a whole chromosome duplication as well as a GCR (Figure 7a). The frequency of whole chromosome duplications in the *hht2* mutants was either not significantly different (*hht2::H3K56G*, Fisher's Exact test; p = 0.289) or slightly reduced (*hht2::H3K56R*, Fisher's Exact test; p = 0.0155) compared to the *rtt109Δ* mutant. We further investigated the distribution of chromosome arm duplications for the GCRs derived from the *hht2* mutant strains to the distribution seen for the wild-type strain. We focused on seven chromosome arm duplications (chrIII-R, IV-R, V-R, VII-R, VIII-L, XII-R, and XVI-R) that shared duplication differences in the *rtt109Δ* and *asf1Δ* single mutants when compared to the distribution observed for the wild-type strain (Figure 7b; Table S2). The profile seen for the *hht2::H3K56G* mutant shared 4 of the 7 changes (Hypergeometric test; p = 0.0187), while the profile seen for the *hht2::H3K56R* mutant shared 5 of the 7 changes (Hypergeometric test; p = 7.72×10^−6^), supporting the view that these two *hht2* mutations are similar to the *rtt109Δ* and *asf1Δ* in regard to their effects on the suppression of GCRs. Overall, these results suggest that the loss of acetylation at histone H3K56 results in high rates of GCRs and whole chromosome duplications.

## Discussion

In this study, we adapted MLPA to identify chromosome arm duplications and deletions, as well as whole chromosome duplications, in order to provide insights into the processes that suppress and promote GCRs in *S. cerevisiae*. Compared to previous methods used to analyze GCRs [7], [11], [12], [14], [15], [20]–[29], this method is rapid, affordable, and of sufficiently high resolution to provide useful structural insights to guide subsequent analysis. We validated the utility of this method by investigating bias in target site selection of Ty1-mediated translocations, *RAD52*-independent Ty1-mediated translocations that appear to occur by HR, and whole chromosome duplications that occur at increased rates in an *rtt109Δ* mutant. This ability to generate structural information for a large numbers of GCRs allowed us to demonstrate the existence of a number of translocation target hotspots and demonstrate that these hotspots likely mediate translocations by different mechanisms. In addition, we obtained evidence for a *RAD52*-independent HR pathway that can promote Ty1-mediated translocations, as well as evidence for an association between whole chromosome duplications and GCRs in an *rtt109Δ* mutant. These results demonstrate that the methods developed here will facilitate future analysis of GCR structures in other mutant backgrounds, which will likely reveal yet other unanticipated aspects of the mechanisms that prevent GCRs.

We previously observed that GCRs isolated in the presence of *Ty912* located on a nonessential arm of chromosome V were almost exclusively associated with a loss of the region of chromosome V-L from the *Ty912* to the left telomere and a duplication of a terminal region of another chromosome arm [12]. In nearly all cases, these GCRs were the products of nonreciprocal translocations mediated by HR between *Ty912* on chromosome V-L and an ectopic Ty element on a target chromosome. Using MLPA probes hybridizing to the telomeric ends of the chromosomes, we identified four chromosome arms (the right arm of chromosome III, the right arm of chromosome V, the left arm of chromosome XIV, and the right arm of chromosome X) that were the target of approximately 70% of the translocations in a wild-type strain and thus appeared to contain hotspots for HR events resulting in *Ty912*-mediated translocations. Detailed analysis using MLPA probes specific to these chromosome arms demonstrated that there were only 6 hotspots that mediated these translocations versus at least 254 potential targets for Ty-mediated translocations in the *S. cerevisiae* genome, and that furthermore, these hotspots were composed of Ty elements.

We considered a number of potential explanations to account for the existence of the observed translocation hotspots; however, no single factor could explain the observed translocation target distributions. First, sequence homology with *Ty912* did not explain the hotspots, as the target Ty elements at the hotspots did not share the highest degree of homology with *Ty912*. Additionally, the target site distribution was relatively unaffected by an *msh2Δ* mutation that increased the efficiency of homeologous recombination, suggesting that the hotspot distribution was not determined by sequence homology. Second, the location of essential genes likely played little or no role in generating the target site distribution. The region of the left arm of chromosome V that is deleted in the GCRs detected by the assay contains only non-essential genes, and all of the translocations detected were non-reciprocal translocations that only duplicated other regions of the genome. Hence, no essential gene was ever observed to be deleted or would be expected to be deleted in this assay. Third, the distribution of centromere- (52.4% of Ty elements) vs. telomere- (47.6% of Ty elements) oriented Ty elements does not explain the distribution of target hotspots as almost all of the chromosome arms contain centromere- and telomere-oriented Ty elements, and the hotspot usage observed was significantly different compared to the distribution of all Ty elements as well as the distribution of only telomere-oriented Ty elements. Furthermore, translocations mediated by centromere-oriented Ty elements, which result in intermediate dicentric translocation chromosomes, were recovered using our assay, and at least 1 of the 6 observed translocation hotspots has been previously demonstrated to mediate the formation of dicentric translocations that undergo additional rounds of rearrangements [12]. Finally, analysis of the data from 4C experiments [36] showed that the spatial proximity of the hotspot targets relative to *Ty912* on the left arm of chromosome V was also unable to explain the hotspots. Thus, target selection seems likely to reflect structural features of either individual Ty elements that are the targets of Ty-mediated translocations, or structural features of the region of the genome in which they reside.

The translocation hotspots on chromosome III-R were located at two previously identified fragile sites (FS1 and FS2) that were induced by down-regulation of the replicative DNA polymerases [14], [15]. Consistent with previous studies [15], [27], [33], each of these fragile sites contains a pair of Tys that were targeted by *Ty912*-mediated translocations, with FS1 containing 2 tandemly repeated Ty1 sequences and FS2 containing a pair of inverted Ty1 sequences separated by a short spacer sequence. The fact that we identified these same two fragile sites using our assay that detects spontaneous rearrangements implies that these sites are also fragile under conditions of normal DNA replication. We previously suggested that *Ty912*-mediated translocations might occur by a mechanism in which a broken chromosome V was repaired by break-induced replication (BIR) in which the *Ty912* on a broken chromosome V promotes strand invasion at the site of ectopic Ty elements and primes DNA synthesis, resulting in copying the terminal region of the target chromosome from the target Ty element to the telomere onto the end of the broken chromosome V [12]. However, the observation that FS1 and FS2 are hotspots for *Ty912*-mediated translocations suggests at least two other possible mechanisms: HR between two DSBs, one on chromosome III-R and one on chromosome V-L, or BIR initiated by a DSB at either FS1 or FS2 that then invades chromosome V at *Ty912* and is followed by loss of the intact chromosome V left by BIR during the selection for the GCR. The fact that another Ty hotspot on chromosome X-R also appears to contain tandem Ty1 elements (*YJRWTy1-1* and *YJRWTy1-2*) (Table 1) suggests that this site might also represent a fragile site, although other there are Ty1 loci in the genome that also contain tandem Ty1 sequences and these did not appear to be translocation target hotspots sites. The hotspot on chromosome XIV-L and the two hotspots on chromosome V-R, one of which has previously been observed as a target of Ty-mediated translocations [9], [12], [26], are not annotated to contain tandem or inverted Ty elements and it is not clear what causes these sites to act as translocation hotspots.

We previously observed that a large proportion of *Ty912*-mediated translocations were mediated by *RAD52*-dependent HR and we obtained genetic evidence that a *RAD51*–dependent HR pathway primarily suppressed Ty-mediated translocations in wild-type strains, whereas a *RAD59*–dependent HR pathway promoted Ty-mediated translocations in the absence of *RAD51*. In agreement with this, we found that a *rad51Δ* mutation increased both the rate of accumulation and the diversity of chromosome arm duplications, and that a *rad59Δ* mutation decreased the rate of most of the individual types of GCRs that occurred in a *rad51Δ* mutant. Interestingly, in the *rad51Δ* mutant, several of the chromosome arms that were duplicated at increased rates did not have full-length Ty1 elements but only contained solo delta elements (chrVII-L, chrIX-L, chrXI-L, and chrXII-L). As *RAD59*-dependent HR promotes SSA between short repeat sequences [50] this raises the possibility that some of the translocation targets that show increased targeting in the *rad51Δ* mutant may reflect increased rates of HR between short repeated sequences mediated by *RAD59*-mediated HR. The majority of the GCRs isolated from *rad51Δ* and *rad59Δ* single mutants and *rad51Δ rad59Δ* double mutant strains were the result of nonreciprocal translocations between *Ty912* and ectopic Tys like those seen in wild-type strains. A striking finding was the almost complete elimination of translocations mediated by the chromosome III-R hotspots in the *rad51Δ* mutant in contrast to the other GCRs, including those mediated by the other translocation hotspots whose rates were increased in the *rad51Δ* mutant and decreased in the *rad51Δ rad59Δ* double mutant. This suggests that structural features of the translocation targets affect the mechanism of translocation. For example, the chromosome III-R hotspot may be a particularly good substrate for BIR given that the translocations mediated by this hotspot were highly dependent on *RAD51*, consistent with prior results [16], [24], [51], whereas the other translocation targets might be more amenable to aberrant repair by *RAD59*-dependent single-stranded annealing (SSA) of broken chromosomes at the site of Ty elements [50], [52], [53].

The results described here also support the idea that Ty-mediated translocations can occur via a *RAD52*-independent HR pathway. Deletion of *RAD52* reduced the rate of Ty-mediated GCRs significantly below the wild-type rate, although not completely to the level observed in a *rad52Δ* mutant in the absence of the *Ty912* element. Consistent with this, a significant proportion of GCRs in a *rad52Δ* mutant with the *Ty912* present appeared to result from *de novo* telomere addition after chromosome breakage. However, there was also a significant fraction of translocations mediated by HR between *Ty912* and ectopic Ty elements in a *rad52Δ* mutant. These Ty-mediated translocations were the same types of translocations seen in wild-type strains. At first glance, our data suggest that the *Ty912*-mediated GCRs in a *rad52Δ* mutant might occur by the same type of *RAD52*-independent recombination seen for other repetitive elements, which is believed to involve SSA followed by half-crossover mechanism [54], [55]. However, simultaneous deletion of both *RAD51* and *RAD59*, or deletion of *RAD1* (which can promote SSA by removal of nonhomologous DNA flaps [56]), in the *rad52Δ* mutant did not reduce the GCR rate further and resulted in distributions of GCRs that were similar to that seen in the *rad52Δ* single mutant. Furthermore, we demonstrated that several of the translocations seen in the *rad52Δ rad51Δ rad59Δ* triple mutant were due to HR between *Ty912* and ectopic Ty elements. This suggests that there might be other mechanisms for promoting recombination or that other nucleases besides Rad1–Rad10 can cleave off non-homologous tails that form during SSA [57], [58].

Previous studies have observed occasional examples of strains containing GCRs and independent whole chromosome duplications [12], [18], [23]. In the present study, we used MLPA to detect whole chromosome duplications in large numbers of independent GCR-containing *rtt109Δ*-derived isolates. We found a statistically significant association of GCRs and whole chromosome duplications in these isolates. Because an *rtt109Δ* mutation caused an increase in the accumulation of whole chromosome duplications, but the presence of a whole chromosome duplication did not increase the GCR rate of an *rtt109Δ* mutant, and because the presence of GCRs did not increase the accumulation of whole chromosome duplications in an *rtt109Δ* mutant, it seems likely that in an *rtt109Δ* mutant, whole chromosome duplications tend to arise with GCRs. *RTT109* encodes a histone H3 lysine 56 acetylase [41], [42] and our analysis demonstrated that the suppression of GCRs and the suppression of whole chromosome duplications associated with GCRs was due to the role of the Rtt109-Asf1 complex in the acetylation of H3K56. The mechanism by which acetylation of H3K56 suppresses GCRs and aneuploidy is currently unclear; however, the observation that *rtt109Δ* mutants have defects in recovery from DNA damage induced checkpoint activation [43], [44], [59], but are not DNA repair defective per se, suggests aberrant recovery from arrest due to the DNA damage that results in GCRs might also result in mis-segregation of chromosomes during mitosis.

## Methods

### DNA isolation, PCR, mutant strain construction, GCR rate calculations, aCGH, and plasmids

General methods have been previously described [7], [11], [12], [60]. Genomic DNA preparations were quantified using a Qubit Fluorometer (Invitrogen). All strains used in this study were derivatives of S288c and are described in Table S12. Plasmids pFX04 and pFX06 [61] were used to create *hht2::H3K56G HHF2* and *hht2:H3K56R HHF2* strains. Ty1 fusion junctions were amplified with Velocity DNA Polymerase (Bioline) using the following protocol: 2 min 98°C initial denaturation step; 25 cycles of 12 sec 98°C denaturation, 30 sec 63.8°C annealing, 3 min 30 sec 72°C extension; final 4 min 72°C extension.

### Multiplex Ligation–dependent Probe Amplification (MLPA): Primer design

Primer design followed the recommendations for synthetic-probes available on the MRC-Holland website (http://www.mrc-holland.com), with the exception that the minimum length differences between amplification products was designed to be 2 base pairs. All primers were purchased and synthesized by ValueGene (http://www.valuegene.com). We designed a total of six sets of primers. Two primer sets were designed to detect copy number variation of different chromosome arms. The set of 32 telomeric probe pairs (Table S1; Figure 1b) was designed to hybridize to unique sequence located between chromosome telomeres and the most distal Ty1 or solo delta elements; four probes (corresponding to the left arm of chromosome II, the right arm of chromosome IV, the right arm of chromosome IX, and the left arm of chromosome XV) were designed to hybridize centromeric to the most distal Ty1 or solo delta elements due to the lack of suitable unique sequence in the preferred region. The set of 32 centromeric probe pairs (Table S9; Figure 1b) was designed to hybridize to unique sequence located centromeric to the Ty1 or solo delta elements closest to the centromere of each chromosome arm. Ty2 elements were represented in this analysis as two independent solo delta elements per Ty2 element as these delta elements contain the majority of the homology between Ty2 and Ty1 elements. We also designed four primer sets capable of pinpointing duplications along whole chromosome arms for chromosomes III-R, V-R, X-R, and XIV-L (Tables S3, S5, S6, S7, respectively). These primer sets generally contained pairs of probes that hybridized immediately telomeric and centromeric to each Ty1 and solo delta element on the pertinent chromosome arm (Figure 3a–3d).

### MLPA: Amplification reaction

All amplification reagents were purchased from MRC-Holland (http://www.mrc-holland.com). The MLPA reaction has been previously described for human genomic DNA [32]. Briefly, probes are hybridized to chromosomal DNA, ligated, and amplified by PCR using universal priming sequences (Figure 1c). Modifications for analysis of *S. cerevisiae* genomic DNA were as follows: 5 ng of template genomic DNA was used instead of 50–100 ng; a 10 min initial 98°C denaturation step was used instead of 5 min; 23 (for chromosome arm specific probe sets) or 25 (for telomeric or centromeric probe sets) PCR cycles were used instead of 35 cycles; and, importantly, the use of thin walled PCR tube strips and plates.

### MLPA: Fragment separation and detection

Fragment separation was carried out as suggested by MRC-Holland on an ABI 3730XL sequencer using POP7 polymer and GS500-LIZ sizing standard (ABI). 8.95 µl of Hi-Di Formamide (ABI) and 0.05 µl of GS500-LIZ sizing standard were mixed with 1 µl of the MLPA PCR product per isolate for a total volume of 10 µl. Then an 82°C heat denaturation step was performed for 2 minutes followed by an incubation step at 4°C for 5 minutes before analysis on the ABI3730XL sequencer instead of the suggested 80°C/4°C steps. Raw peak data for each run was obtained via the GeneMapper software from ABI.

### MLPA: Data analysis

Data analysis was performed using Python (version> = 2.5) and as described by MRC-Holland (http://www.mrc-holland.com).

#### Analysis of telomeric and centromeric probe values

Briefly, normalized probe values were generated for each sample in a run by dividing raw probe values by the sum of the total area for all probes in a sample. Next, the value for each probe in the sample was normalized to the average of the control samples' respective normalized probe values by dividing the sample's normalized probe value by the average of the pertinent normalized probe values of the control samples in a run. Copy number was assigned by rounding this final normalized value to the nearest integer.

#### Analysis of chromosome arm specific probe values

Analysis for specific chromosome arm probe sets was performed as above, except for the following modification. Normalized probe values for each sample in a run were generated by dividing the value for every probe in the sample by the most centromeric probe value, on the assumption that the most centromeric probe was not duplicated. The rest of the analysis was performed as described above.

### Calculation of the ratio of observed versus expected rates for the duplication of each chromosomal arm

The observed rate of the duplication of each chromosomal arm for a strain was taken to be the total Can^R^ 5FOA^R^ rate of the strain multiplied by the fraction of GCRs associated with the duplication of that chromosomal arm. The expected rate of a test strain relative to a control strain was calculated by multiplying the observed duplication rate for each chromosome arm in the control strain by a scaling constant. The scaling constant was the sum of the rates of the duplications of all chromosome arms in the test strain divided by the sum of the rates of the duplications of all chromosome arms in the control strain. This scaling constant was very similar to the ratio of Can^R^ 5FOA^R^ rates of the test and control strains for those strains dominated by GCRs associated with chromosome arm duplications, but properly handles mutations like *rad52Δ* which results in a substantial fraction of GCRs lacking chromosome arm duplications. In cases where no duplications for an individual chromosome arm were found in the control strain, then the expected rate was estimated as the upper limit of the duplication rate of that chromosomal arm (the total Can^R^ 5FOA^R^ rate divided by the number of duplications). In cases where no chromosome arm duplication was found in the test strain, then the upper limit of the rate was used if it was less than the expected rate, otherwise the ratio of observed vs. expected rates was set to 1. In cases where no duplications for an individual chromosome arm were found in both the test and control strain, then the ratio of observed vs. expected rates was set to 1.

### Statistics

R (version≥2.11.1) was used to calculate p-values for Wilcoxon rank-sum tests, Fisher exact tests, the Monte Carlo sampling of multinomial distributions, the Hypergeometric test and the Binomial calculation. Ninety-five percent confidence intervals for the median GCR rates were calculated using a two-sided nonparametric test (http://www.math.unb.ca/~knight/utility/MedInt95.htm). Individual chromosome arm duplication rates were considered to be statistically significantly different from the bulk rate of a mutant if the calculated individual chromosome arm duplication rates fell outside the transformed 95% confidence interval of the bulk fold rate of the mutant strain.

## Supporting Information

Figure S1 Distribution of telomere-oriented Ty1 and solo delta elements on each chromosome arm.
(PDF)

Figure S2 aCGH analysis of *rad52Δ* GCR-containing strains. All isolates had the *rad52Δ* mutation. Two isolates (I15 & I18) had only a chromosome V-L deletion. Five isolates (I16, I17, & I19–I21) had a deletion from *Ty912* to *TEL05L* on chromosome V and a duplication on another chromosome arm bordered by a Ty and a telomere. Deletions are depicted by absolute probe intensities. Duplications are depicted by log2 ratios of intensities.
(PDF)

Figure S3 Comparison of chromosome arm duplication rates in various HR deficient strains to those from a *rad52Δ* mutant strain. The chromosome arm duplication distributions of the different double mutants were highly similar to that of the *rad52Δ* mutant, indicating that the *rad52Δ* mutation was epistatic to the other mutations tested.
(PDF)

Figure S4 Log2 ratio of observed vs. expected chromosome arm duplication rates of *asf1Δ*, *rlf2Δ*, and *asf1Δ rlf2Δ* mutant strains vs. wild type. The distribution of chromosome arm duplications for the *asf1Δ rlf2Δ* strain appears to be additive and composed of different components of the chromosome arm duplication distribution seen in the *asf1Δ* and *rlf2Δ* single mutants.
(PDF)

Table S1 Sequences of the oligonucleotide primers for the telomeric MLPA probe set. The chromosome arm, the gene/site that the primers are homologous to and the primer sequences are listed.
(XLSX)

Table S2 The number of chromosome arm deletions and chromosome arm duplications observed. The relevant strain genotype, the strain identification number, the GCR rate for the strain, the number of times each solo chromosome arm deletion or chromosome arm duplication was observed, and the number of independent GCRs analyzed are indicated.
(XLSX)

Table S3 Sequences of the oligonucleotide primers for the chromosome III-R MLPA probe set. The Ty element analyzed, the position of the probe centromeric or telomeric to the element, the gene/site that the primers are homologous to and the primer sequences are listed.
(XLSX)

Table S4 Analysis of 4C data indicates that the *Ty912* is not adjacent to the 6 different translocation hotspots inside the nucleus. The locus names of *Ty912* and the 6 different translocation hotspots, their chromosome arm positions, the nucleotide coordinates of these positions and the nucleotide coordinates of the region +10 kb to −10 kb surrounding these positions are indicated. These positions were then analyzed using the indicated 4C data to identify the number and coordinates of the HindIII and EcoRI fragments containing either the individual loci or the region +/−10 kb surrounding these loci and to determine how many of the potential combinations of *Ty912* loci fragments and fragments representing each reported hotspot locus had significant interactions. The results are indicated as # of hits (interactions) and total possible # of hits (interactions).
(XLSX)

Table S5 Sequences of the oligonucleotide primers for the chromosome V-R MLPA probe set. The Ty element analyzed, the position of the probe centromeric or telomeric to the element, the gene/site that the primers are homologous to and the primer sequences are listed.
(XLSX)

Table S6 Sequences of the oligonucleotide primers for the chromosome X-R MLPA probe set. The Ty element analyzed, the position of the probe centromeric or telomeric to the element, the gene/site that the primers are homologous to and the primer sequences are listed.
(XLSX)

Table S7 Sequences of the oligonucleotide primers for the chromosome XIV-L MLPA probe set. The Ty element analyzed, the position of the probe centromeric or telomeric to the element, the gene/site that the primers are homologous to and the primer sequences are listed.
(XLSX)

Table S8 Analysis of 11 independent GCR containing isolates from a *rad52Δ* +Ty912 strain using MLPA and aCGH. The isolate name, relevant strain genotype, strain number, techniques used to analyze the isolate, the events observed and the translocation target are all indicated. The GCRs observed are either described using ISCN-style rules for describing chromosomal rearrangements or, where less information was available, are described by listing the observed chromosome arm deletions and duplications as “−” chromosome arm # R or L or “+” chromosome arm # R or L.
(XLSX)

Table S9 Sequences of the oligonucleotide primers for the centromeric MLPA probe set. The chromosome arm, the gene/site that the primers are homologous to and the primer sequences are listed.
(XLSX)

Table S10 MLPA analysis of 49 independent cultures obtained by fluctuation analysis of an *rtt109Δ* mutant. Each culture was plated on both YPD and GCR selective plates, and then one colony from each plate was analyzed for the presence of chromosome arm duplications or deletions and whole chromosome duplications using MLPA. The culture/isolate name, the events observed in each YPD plate isolate and each GCR selective plate isolate, and the GCR rate of each culture are indicated. Whole chromosome duplications are labeled “+” chromosome # and chromosome arm duplications or deletions are labeled as “+” or “−” chromosome # R or L.
(XLSX)

Table S11 MLPA analysis of 21 independent spore clones isolated from an *rtt109Δ*+chromosome VIII diploid. Twenty-one independent spore clones were obtained by sporulation of an *rtt109Δ*+chromosome VIII diploid. Each spore clone was grown in liquid YPD media and then the culture was plated on both YPD and GCR selective plates, and then one colony from each plate was analyzed for the presence of chromosome arm duplications, deletions, and whole chromosome duplications using MLPA. The culture/isolate name, the events present in each initial spore clone, the events observed in the YPD plate isolate and each GCR selective plate isolate and the GCR rate of each culture are indicated. Whole chromosome duplications are labeled “+” chromosome # and chromosome arm duplications or deletions are labeled as “+” or “−” chromosome # R or L.
(XLSX)

Table S12 The strains used in this study. The strain number and the complete genotype of each strain used are listed.
(XLSX)
