# Supplementary figures and images for: Rapid Analysis of Saccharomyces cerevisiae Genome Rearrangements by Multiplex Ligation–Dependent Probe Amplification
Source: PLoS Genet. 2012 Mar 1;8(3):e1002539. doi: 10.1371/journal.pgen.1002539 (PMC3291544; doi:10.1371/journal.pgen.1002539)

**Figure S1**

**Expected Frequency of  
Telomere Oriented Ty1 and Delta  
Elements**

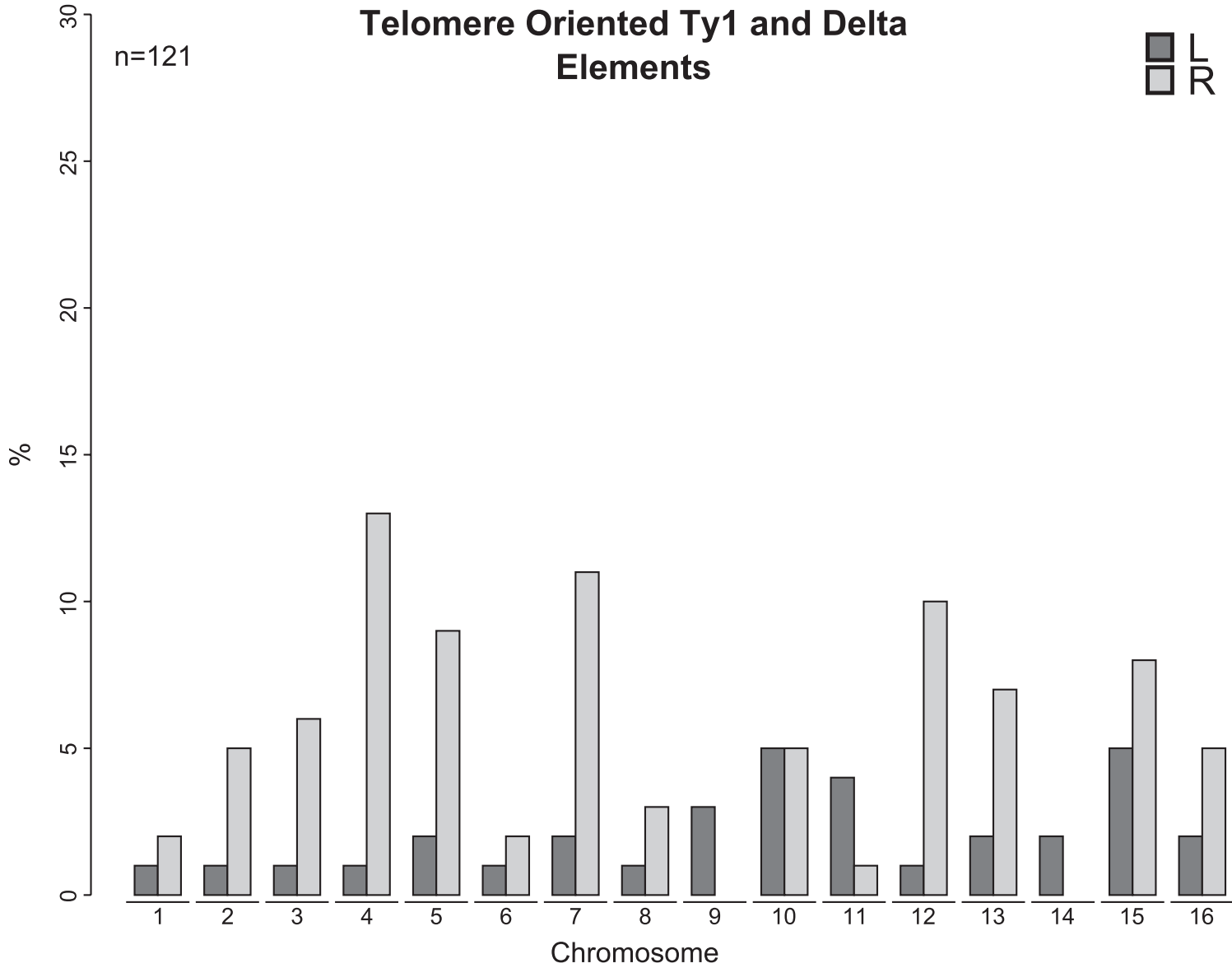

Supplement: Figure S1 — Distribution of telomere-oriented Ty1 and solo delta elements on each chromosome arm. (PDF) [file pgen.1002539.s001.pdf]

**Figure S2**

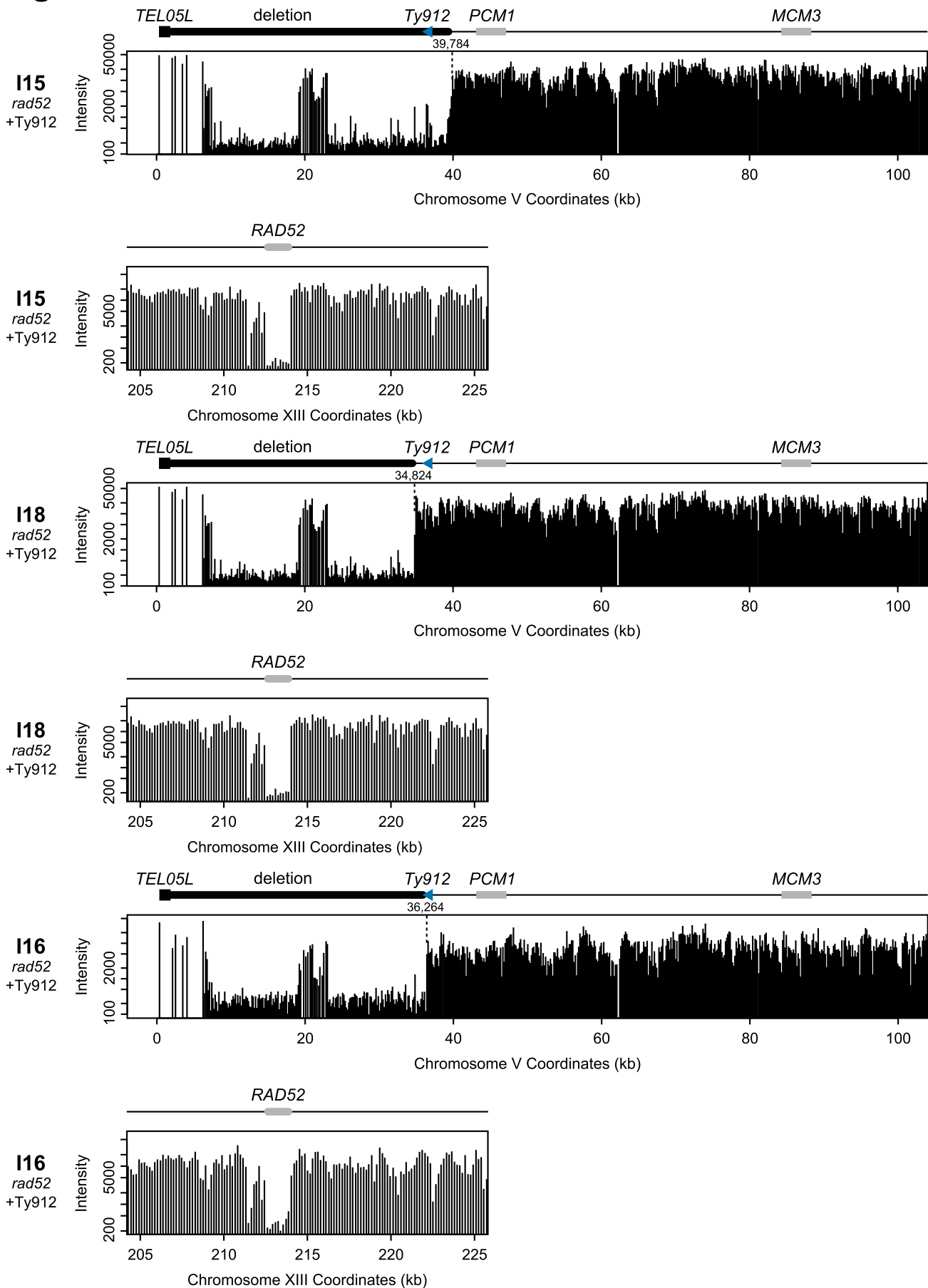

Figure S2 (continued)

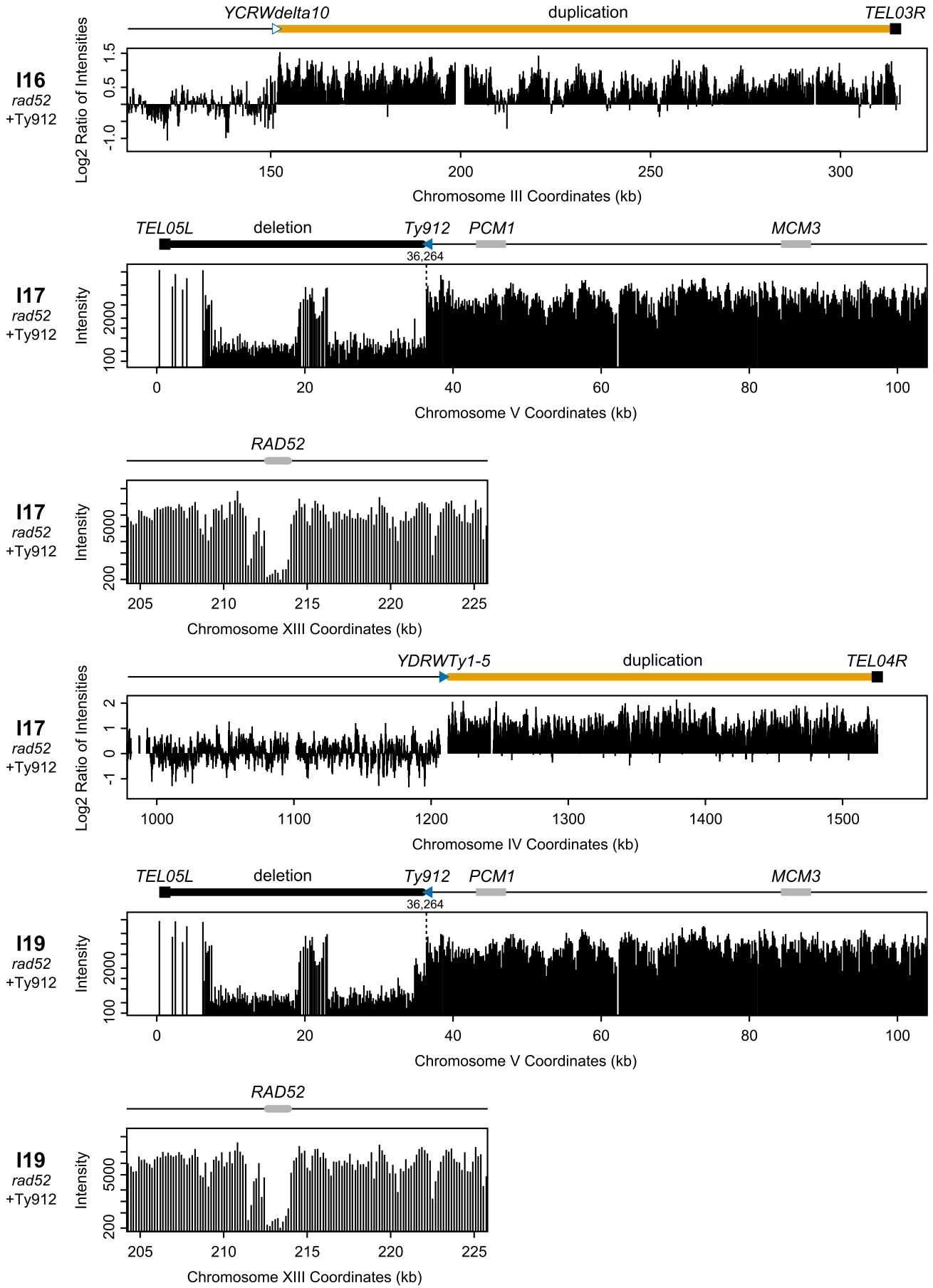

# Figure S2 (continued)

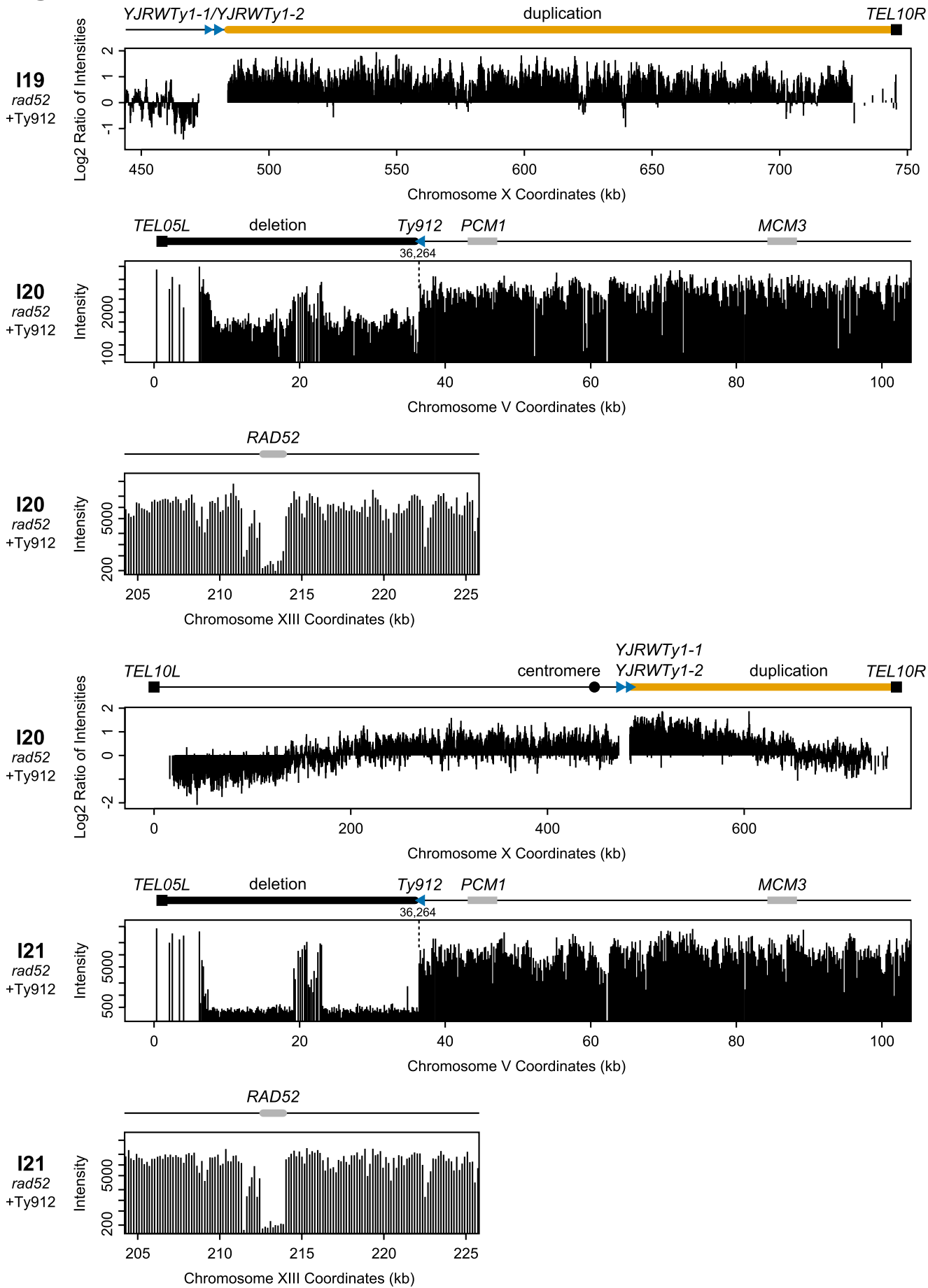

Figure S2 (continued)

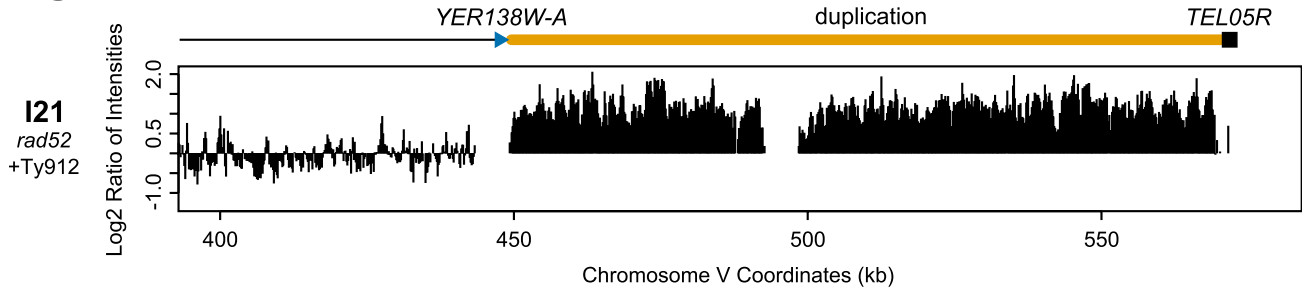

Supplement: Figure S2 — aCGH analysis of rad52Δ GCR-containing strains. All isolates had the rad52Δ mutation. Two isolates (I15 & I18) had only a chromosome V-L deletion. Five isolates (I16, I17, & I19–I21) had a deletion from Ty912 to TEL05L on chromosome V and a duplication on another chromosome arm bordered by a Ty and a telomere. Deletions are depicted by absolute probe intensities. Duplications are depicted by log2 ratios of intensities. (PDF) [file pgen.1002539.s002.pdf]

Figure S3

*rad52Δ rad51Δ*, *rad52Δ rad59Δ*, *rad52Δ rad1Δ* vs. *rad52Δ*

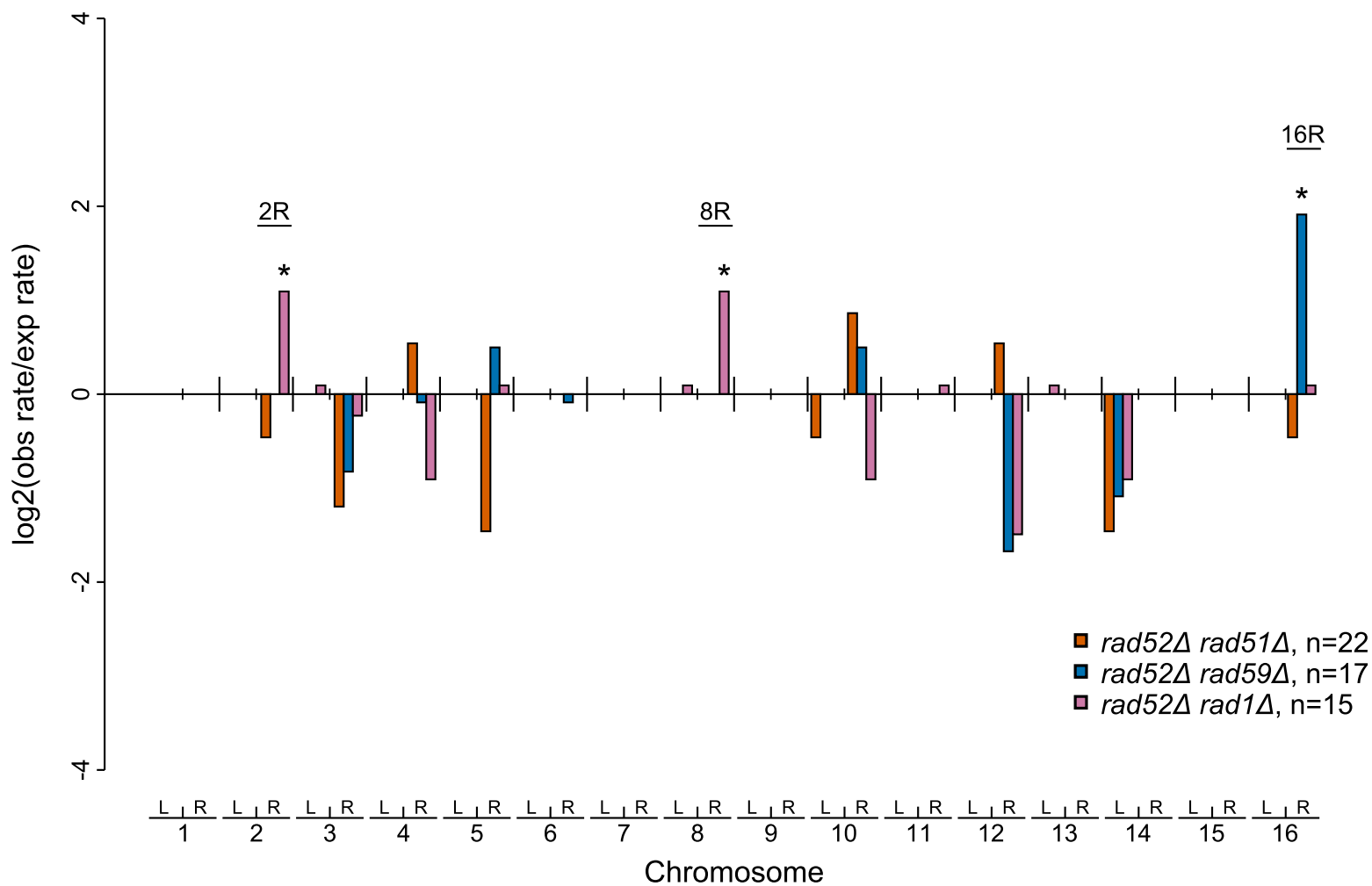

Supplement: Figure S3 — Comparison of chromosome arm duplication rates in various HR deficient strains to those from a rad52Δ mutant strain. The chromosome arm duplication distributions of the different double mutants were highly similar to that of the rad52Δ mutant, indicating that the rad52Δ mutation was epistatic to the other mutations tested. (PDF) [file pgen.1002539.s003.pdf]

Figure S4

*asf1Δ, rlf2Δ, asf1Δ rlf2Δ* vs. WT

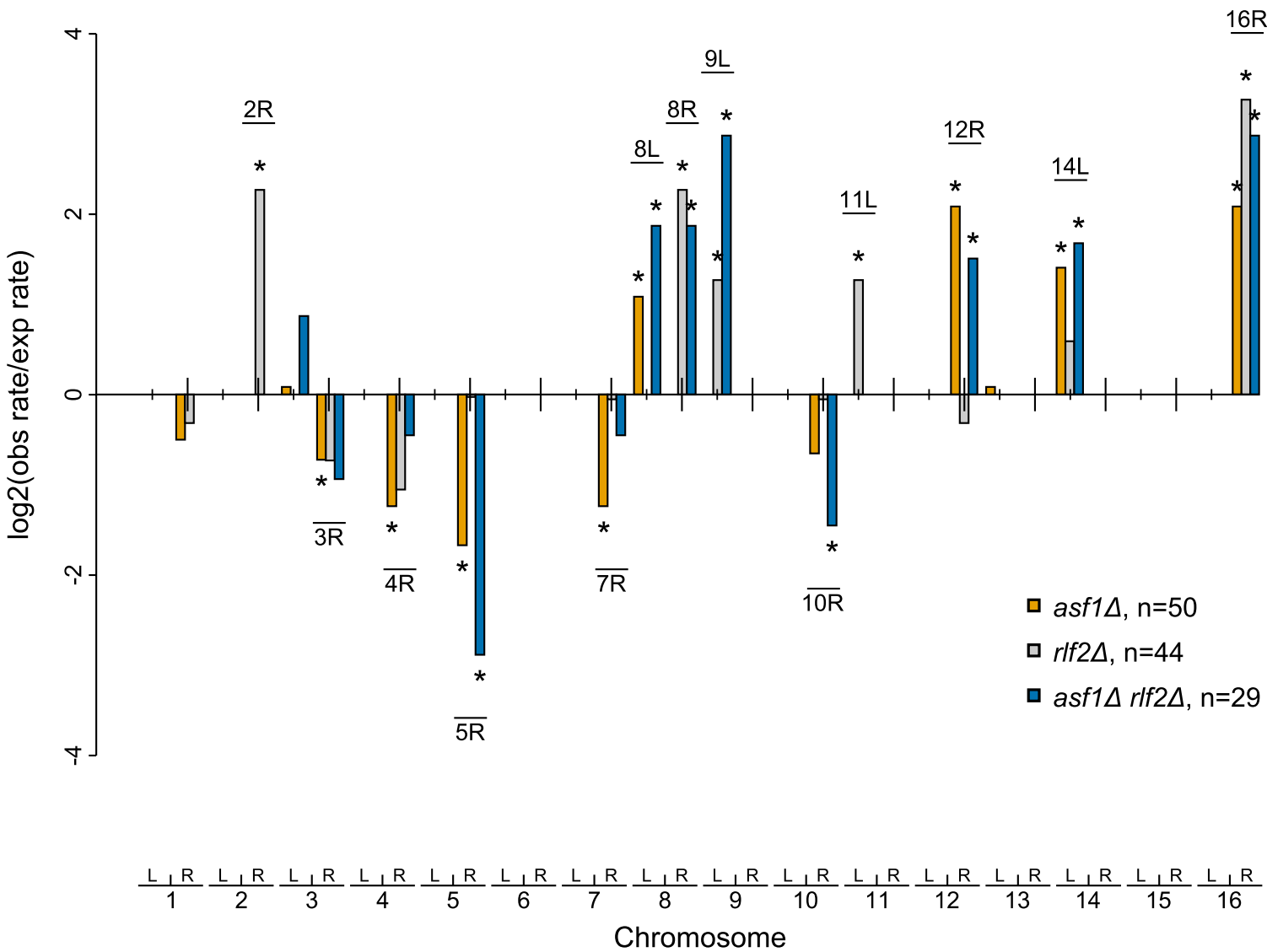

Supplement: Figure S4 — Log2 ratio of observed vs. expected chromosome arm duplication rates of asf1Δ, rlf2Δ, and asf1Δ rlf2Δ mutant strains vs. wild type. The distribution of chromosome arm duplications for the asf1Δ rlf2Δ strain appears to be additive and composed of different components of the chromosome arm duplication distribution seen in the asf1Δ and rlf2Δ single mutants. (PDF) [file pgen.1002539.s004.pdf]
